# Unsaturation of Very-Long-Chain Ceramides Protects Plant from Hypoxia-Induced Damages by Modulating Ethylene Signaling in *Arabidopsis*

**DOI:** 10.1371/journal.pgen.1005143

**Published:** 2015-03-30

**Authors:** Li-Juan Xie, Qin-Fang Chen, Mo-Xian Chen, Lu-Jun Yu, Li Huang, Liang Chen, Feng-Zhu Wang, Fan-Nv Xia, Tian-Ren Zhu, Jian-Xin Wu, Jian Yin, Bin Liao, Jianxin Shi, Jian-Hua Zhang, Asaph Aharoni, Nan Yao, Wensheng Shu, Shi Xiao

**Affiliations:** 1 State Key Laboratory of Biocontrol and Guangdong Provincial Key Laboratory of Plant Resources, School of Life Sciences, Sun Yat-sen University, Guangzhou, China; 2 State Key Laboratory of Agrobiotechnology, School of Life Sciences, The Chinese University of Hong Kong, Hong Kong, China; 3 School of Life Sciences and Biotechnology, Shanghai Jiao Tong University, Shanghai, China; 4 Department of Plant Sciences, Weizmann Institute of Science, Rehovot, Israel; National University of Singapore and Temasek Life Sciences Laboratory, SINGAPORE

## Abstract

Lipid remodeling is crucial for hypoxic tolerance in animals, whilst little is known about the hypoxia-induced lipid dynamics in plants. Here we performed a mass spectrometry-based analysis to survey the lipid profiles of *Arabidopsis* rosettes under various hypoxic conditions. We observed that hypoxia caused a significant increase in total amounts of phosphatidylserine, phosphatidic acid and oxidized lipids, but a decrease in phosphatidylcholine (PC) and phosphatidylethanolamine (PE). Particularly, significant gains in the polyunsaturated species of PC, PE and phosphatidylinositol, and losses in their saturated and mono-unsaturated species were evident during hypoxia. Moreover, hypoxia led to a remarkable elevation of ceramides and hydroxyceramides. Disruption of ceramide synthases LOH1, LOH2 and LOH3 enhanced plant sensitivity to dark submergence, but displayed more resistance to submergence under light than wild type. Consistently, levels of unsaturated very-long-chain (VLC) ceramide species (22:1, 24:1 and 26:1) predominantly declined in the *loh1*, *loh2* and *loh3* mutants under dark submergence. In contrast, significant reduction of VLC ceramides in the *loh1-1 loh3-1* knockdown double mutant and lacking of VLC unsaturated ceramides in the *ads2* mutants impaired plant tolerance to both dark and light submergences. Evidence that C24:1-ceramide interacted with recombinant CTR1 protein and inhibited its kinase activity *in vitro*, enhanced ER-to-nucleus translocation of EIN2-GFP and stabilization of EIN3-GFP *in vivo*, suggests a role of ceramides in modulating CTR1-mediated ethylene signaling. The dark submergence-sensitive phenotypes of *loh* mutants were rescued by a *ctr1-1* mutation. Thus, our findings demonstrate that unsaturation of VLC ceramides is a protective strategy for hypoxic tolerance in *Arabidopsis*.

## Author Summary

Hypoxia is one of the most important abiotic stresses of worldwide concern that determines the crop productivity and the natural distribution of plant species. Flooding events such as root waterlogging and submergence strongly affect diffusion of gasses into plant cells, which eventually leads to hypoxia and carbohydrate shortages in terrestrial plants. The gaseous hormone ethylene is considered as the primary determinant of hypoxia response in plants. In particular, the Group VII ethylene-responsive factors (ERFs) have been demonstrated to be master regulators for oxygen sensing through an N-end rule protein degradation mechanism. Recent investigations have suggested that lipid molecules such VLC ceramides may play crucial roles in hypoxia signaling in both animal and plant cells. Here, we identified the unsaturated VLC ceramide species including C22:1-, C24:1- and C26:1-Cers which were essential for hypoxia response by interacting with CTR1 protein, inhibiting its kinase activity, and modulating subsequent ethylene signaling in *Arabidopsis*. The dynamic unsaturation of VLC ceramides is likely to serve as a novel protective strategy for enhancing plant tolerance to the frequent environmental stresses, including flooding.

## Introduction

Lipids are essential constituents of plant cells that provide both the structural basis for cell membranes and an energy source for cellular metabolism [1,2]. Recently, considerable attention has been given to the function of lipids as mediators of various biological activities including growth, development and response to biotic and abiotic stresses [1,2]. It is increasingly apparent that the carbon length and saturation of fatty acids are altered in plants in response to environmental cues. For example, significant increases in unsaturated fatty acids occur when plants are adversely stressed [3,4]. Moreover, a wide range of molecules including very-long-chain fatty acids (VLCFAs) and their derivatives such as sphingolipids and cuticular lipids, play indispensable roles in regulation of plant stress responses [5,6].

In plants, *de novo* synthesis of VLCFAs originates from the C16 and C18 saturated fatty acids which are synthesized in the plastids. Subsequently, distinct chain lengths of fatty acids are subjected to elongation by the fatty acid elongase complex in the endoplasmic reticulum (ER) membrane [7]. VLCFAs are direct precursors for biosynthesis of cuticular lipids and sphingolipids; the latter act as major components of the plasma membrane and play key roles in intracellular activities as well as diverse signaling pathways [8,9]. Structurally, the sphingolipids are composed of a polar head group, a sphingoid long-chain base (LCB) and an amide-linked fatty-acyl chain. Among these components, the fatty acid chains vary in length from 16 to 26 carbon atoms, which can be either saturated or unsaturated with a *cis*-ω9 double bond [10]. The LCB components of sphingolipids are derived from the amino acid serine and palmitoyl-CoA in the ER by serine palmitoyltransferase to produce 3-ketosphinganine, which in turn is reduced to form d18:0 sphinganine by an NADPH-dependent 3-ketosphinganine reductase [7,10]. Ceramide is subsequently assembled by acylating sphinganine to an acyl-CoA or free fatty acid with acyl-chain lengths of C16 to C26. Alternatively, ceramides can be formed via a salvage pathway, where ceramides are released from complex sphingolipids such as glycosylceramides and glycosyl-inositol-phosphoceramides [7,10]. Thus, ceramides serve as both intermediates for turnover of sphingolipids and backbones for synthesis of more complex sphingolipids *in planta*.

In *Arabidopsis thaliana*, three genes encode ceramide synthases essential for ceramide biogenesis, namely *LOH1*, *LOH2* and *LOH3* [7,10,11]. The levels of C16 ceramides in *loh2* mutants are almost undetectable, whilst the levels of ceramides with VLCFAs are depleted in the *loh1 loh3* double mutant [12,13], suggesting that these three LOHs are specific for synthesis of distinct acyl-chain lengths of ceramides. Notably, the rosettes of *loh1* single mutants display spontaneous cell death in short-day conditions [13], whereas the *loh1 loh3* double mutant shows lethality in early seedling development [12]. The abundance of sphingolipid species is largely dependent on the structural variation of LCB and fatty acyl chain, as well as their modifications including hydroxylation and desaturation. Moreover, increasing evidence reveals the functional significance of LCB and fatty acid (FA) hydroxylation and desaturation in plant cells [11]. For example, the deletion of C-4 hydroxylases in the *Arabidopsis sbh1 sbh2* double mutant results in a deficiency of trihydroxy LCBs and a dwarf phenotype [14]. In contrast, the *sld1 sld2* double mutant or transgenic lines of LCB Δ8 desaturases shows altered tolerance to low temperature and aluminum toxicity, respectively [15,16]. Meanwhile, ceramide FA hydroxylation is catalyzed by the cytochrome *b*_*5*_-fusion enzymes FAH1 and FAH2. The *fah1 fah2* double mutant displays increased levels of ceramides and salicylic acid, as well as enhanced resistance to the biotrophic pathogen *Golovinomyces cichoracearum* [17]. However, the significance of ceramide FA desaturation remained unknown until recent identification of an acyl-CoA desaturase, ADS2, in *Arabidopsis* [18]. The *ads2* mutant has a significant reduction in the VLC-CoAs and a decline in unsaturated sphingolipids but the biological function of this alteration awaits further investigation.

Hypoxia is one of the most important abiotic stresses that affects the growth and yield of plants. Flooding, including root waterlogging and complete submergence of plants, leads to a decline in the available oxygen, and thus affects physiological activities and plant growth [19–21]. Ethylene is considered to be the primary determinant in plant response to hypoxia [20–22]. Recently, the Group VII ethylene-responsive factors (ERFs) have been demonstrated to be master regulators for oxygen sensing [23–25]. Specifically, one ERF transcription factor, RAP2.12, interacts with the plasma membrane-anchored acyl-CoA binding proteins (ACBP1 and ACBP2) under normoxia [24]. Upon hypoxic stress, RAP2.12 dissociates from the plasma membrane and accumulates in the nucleus to activate transcription of hypoxia-responsive genes [24]. Given the diverse cellular functions of plant ACBPs in lipid metabolism and stress responses [2], it is conceivable that lipids or lipid signaling may play a crucial role in plant response to hypoxic stress.

Recent studies in mammals have revealed that in response to hypoxia, tumor cells promote their survival and growth by scavenging unsaturated fatty acids from lysophospholipids, a process which is independent of the *de novo* lipogenesis pathway [26]. Similarly, dark anoxia induces substantial degradation of FAs and accumulation of unsaturated triacylglycerols (TAGs) in cells of *Chlamydomonas reinhardtii* [27]. In *Caenorhabditis elegans*, loss of the ceramide synthase gene *HYL-1* (*hyl-1*) results in increased resistance to anoxia, whereas deletion of the homologous gene *HYL-2* (*hyl-2*) attenuates the anoxic tolerance of *C*. *elegans* compared with normal worms [28]. Given that synthesis of C20 to C22 ceramides relies on HYL-2, whereas formation of C24 to C26 depends mainly on HYL-1, it appears that the *in vivo* homeostasis of VLC species of ceramides is important for the differential susceptibility of *C*. *elegans* to anoxia [28,29]. Moreover, the endogenous level of dihydroceramides (DHCs) is remarkably increased in mammalian cells exposed to various hypoxic conditions, which are rapidly converted to ceramides by the DHC desaturase (DEGS) after re-oxygenation [30]. In mammals, stress-induced ceramides can specifically bind to the cysteine-rich CR1 domain of protein kinase Raf-1, leading to dynamic modulation of Raf kinase activity and subsequent activation of the MAPK cascade [31,32]. Overall, these findings demonstrate that the adaptive remodeling of lipid metabolism is a necessary process for higher organisms to respond to hypoxic stress, and such a process is likely to be conserved across eukaryotic species.

In this study, we performed a comprehensive mass spectrometry-based analysis to investigate hypoxia-induced lipid remodeling in *Arabidopsis*. In particular, we observed a significant accumulation of ceramides in *Arabidopsis* upon hypoxia under both light and dark submergence conditions. Furthermore, our results demonstrate that unsaturation of VLC-ceramides is likely to be a protective mechanism to hypoxia-stressed plants by modulating ethylene signaling.

## Results

### Expression patterns of lipid metabolism genes under hypoxic stress

Previous investigations using a microarray approach have been performed extensively to evaluate the transcriptome profiles in *Arabidopsis* response to anoxia or hypoxia [33–38]. In these reports, *Arabidopsis* seedlings were treated with either low oxygen (3% O_2_ and 97% N_2_) or complete submergence under constant darkness, in order to induce a severe and rapid onset of anoxia/hypoxia responses in the plants. Hence, all the current available expression data were obtained from *Arabidopsis* seedlings exposed to anoxic/hypoxic or submergence conditions for less than 24 h. In this study, we intended to establish a method that may reflect the natural situation, *i*.*e*., by fully submerging the *Arabidopsis* seedlings under light conditions. Preliminary data showed that in contrast to the almost complete death upon submergence under constant darkness within 3 d, *Arabidopsis* could endure light submergence stress for up to 10 d. The transcripts of hypoxia marker genes such as *ADH1*, *PDC1* and *HUP09* in 4-week-old seedling rosettes were upregulated at 48 and 72 h after light submergence treatment (S1 Fig). Therefore, a time point of 48 h upon light submergence exposure was chosen for microarray analysis to further investigate the transcription profiles of *Arabidopsis* rosettes in response to hypoxic stress.

**Fig 1:**
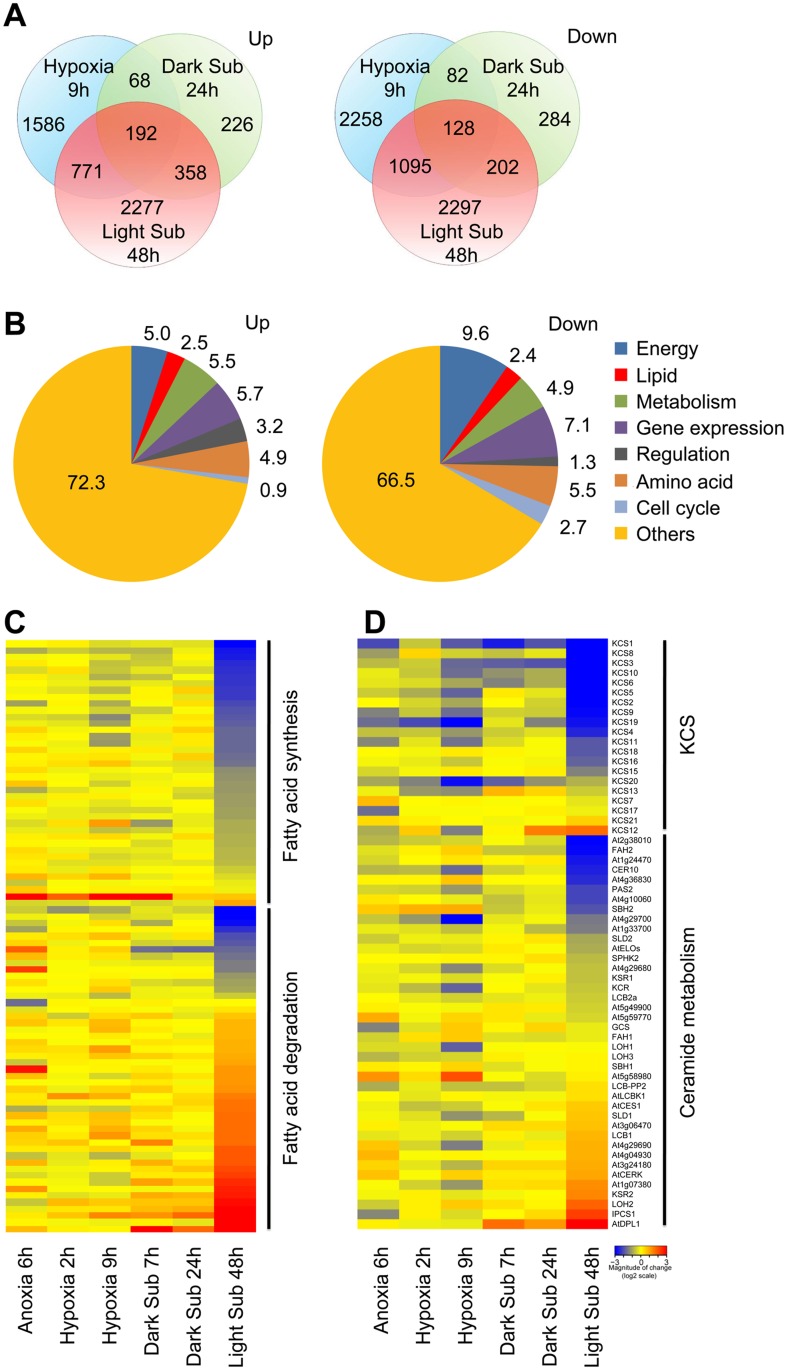
Differentially expressed genes involved in lipid metabolism under hypoxic stress. (A) Venn diagram showing overlapping analysis of the upregulated and downregulated DEGs in the 9-h Hypoxia, 24-h dark submergence (Dark Sub) and 48-h light submergence (Light Sub) treatments. The data of hypoxia and dark submergence treatments were exported from Gene Expression Omnibus database choosing 9-h hypoxia in GSE9719 [78], and 24-h dark submergence in GSE24077 [36]. The Venn diagrams were drawn with the “VennDiagram” package for R. (B) Functional annotation of 3,598 upregulated and 3,722 downregulated genes after 48-h light submergence treatment. Differentially expressed genes (DEGs) were identified by GeneSpring 12.6 with a 1.5-fold change and *P* < 0.05 cutoffs. (C) Hierarchical cluster analysis applied to the 89 DEGs in the fatty acid biosynthesis and degradation in the selected anoxia, hypoxia, and submergence stresses (Anoxia 6h, Hypoxia 2h, Hypoxia 9h, Dark Sub 7h, Dark Sub 24h and Light Sub 48h). The data for anoxia, hypoxia and dark submergence treatments were exported from Gene Expression Omnibus database choosing 6-h anoxia in GSE2133 [77], 2-h and 9-h hypoxia in GSE9719 [78], and 7-h and 24-h dark submergence in GSE24077 [36]. The transcriptional profiles of relative gene expression values (log2 scale of microarray value) were analyzed using the heatmap command of the R language. Red and blue colors represent upregulated and downregulated genes, respectively. (D) Hierarchical cluster analysis applied to the 60 DEGs in the sphingolipid pathway in the selected anoxia, hypoxia and submergence stresses (Anoxia 6h, Hypoxia 2h, Hypoxia 9h, Dark Sub 7h, Dark Sub 24h and Light Sub 48h).

The transcriptomic analysis identified in a total of 7,320 genes differentially expressed more than 1.5 fold, of which 5,617 genes had greater than 2-fold and 3,528 genes had greater than 3-fold changes in expression in the 48-h light submergence-treated seedlings. Among them, 3,598 genes were upregulated and 3,722 genes were downregulated as compared to control samples (Fig 1A and S1 Table). In comparison to the published data with short-time dark submergence or dark hypoxia/anoxia treatments, the number of 48-h light submergence-responsive genes was significantly higher than those of 9-h dark hypoxia and 24-h dark submergence treatments (Fig 1A). Moreover, only 192 upregulated and 128 downregulated genes were shared in all of these treatments (Fig 1A). Notably, light submergence induced significant changes of mRNA abundance in the genes involved in lipid biosynthesis and metabolism, as indicated by functional annotation analysis (Fig 1B). Transcript levels of 89 fatty acid pathway genes were significantly altered by 48-h light submergence (Fig 1C). The hierarchical clustering showed that 48-h light submergence treatment repressed the transcripts of genes in fatty acid synthesis, but significantly induced transcripts of genes in fatty acid degradation pathway(s) (Figs 1C, S2A and S2B and S2 Table). By contrast, this difference was not evident in the dark anoxia/hypoxia or dark submergence treatments (Fig 1C). Furthermore, the transcripts of several members of the KCS gene family that encodes 3-ketoacyl-CoA synthase catalyzing the initial condensation reaction in VLCFA synthesis were repressed significantly by 48-h light submergence, but most of them remained unchanged under dark anoxia/hypoxia or dark submergence at the short-time points (Figs 1D, S2E and S3 Table). Additionally, many genes involved in metabolism of sphingolipids were differentially expressed during 48-h light submergence (Figs 1D, S2G and S3 Table). In particular, the transcript levels of *KSR2*, *LOH2*, *IPCS1* and *DPL1*, which encode enzymes essential for synthesis and catabolism of ceramides and LCBs, were significantly induced under light submergence conditions (Figs 1D and S2G).

Among the 38 light submergence-responsive genes involved in cuticular lipid metabolism, two genes (At3g49210 and At5g12420) encoding putative wax ester synthases were significantly upregulated, whereas the expression of a different wax ester synthase and diacylglycerol acyltransferase, *WSD1*, was repressed by 48-h light submergence (S2E and S2F Fig and S4 Table). In contrast to their slight up- or down-regulation in the short-time anoxia/hypoxia or dark submergence treatments, four genes (CYP96A1, A3, A4 and A12) encoding mid-chain alkane hydroxylases (S2E and S2F Fig) and three genes (*DGK6*, *PIPLC1* and *GPAT3*) in glycerolipid metabolism (S2C and S2D Fig and S5 Table), were significantly repressed by 48-h light submergence. Taken together, our microarray data reveal that the transcripts of genes in lipid metabolism were substantially affected by the 48-h light submergence treatment.

### Changes of lipid molecular species in *Arabidopsis* rosettes upon light submergence exposure

**Fig 2:**
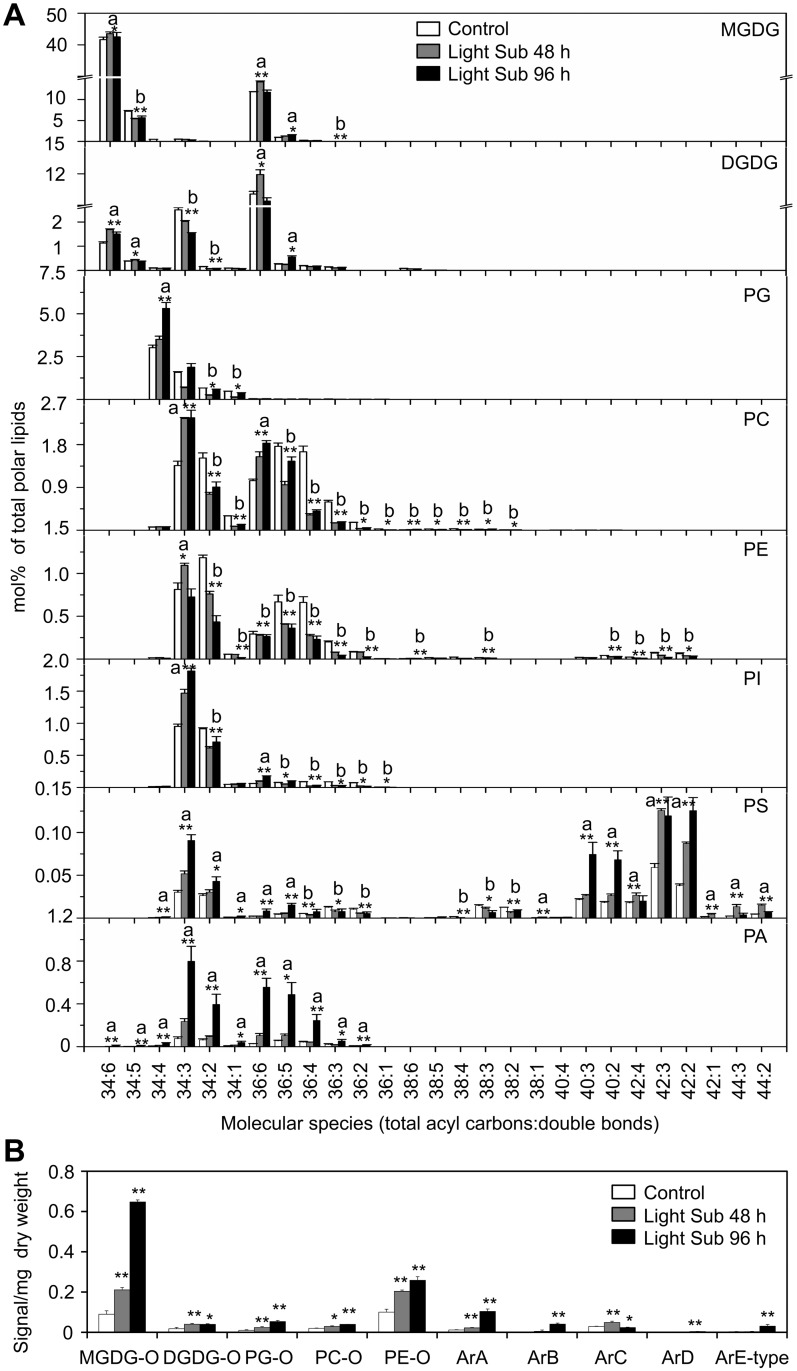
Compositions of phospholipids and oxidized lipids in *Arabidopsis* rosettes upon 48-h and 96-h hypoxia exposure. (A) Contents of different molecular species of membrane lipids (MGDG, DGDG, PG, PC, PE, PI, PS and PA) in 4-week-old wild-type *Arabidopsis* before treatment (Control) and at 48 h (Light Sub48 h) and 96 h (Light Sub 96 h) after light submergence treatment. (B) Amounts of oxidized membrane lipid species (MGDG-O, DGDG-O, PG-O, PC-O and PE-O) as well as complex arabidopsides (ArA, ArB, ArC, ArD and ArE-type) in 4-week-old wild-type *Arabidopsis* before treatment (Control) and at 48 h (Light Sub 48 h) and 96 h (Light Sub 96 h) after light submergence treatment. a, indicates value of Light Sub 48 h or Light Sub 96 h higher than that of Control; b, indicates value of Light Sub 48 h or Light Sub 96 h lower than that of Control (*P<0.05 or **P<0.01 by Student’s *t*-test). Values are the means ±SD (*n* = 4). Control refers to samples harvested from plants grown under the same conditions before treatment.

To investigate the potential role of lipids in hypoxia response, lipid profiles of *Arabidopsis* rosettes following light submergence exposure were analyzed by electrospray ionization–tandem mass spectrometry (ESI-MS/MS). Compared to the seedlings grown under normal growth conditions, significant changes were observed in the membrane lipid contents of *Arabidopsis* rosettes after light submergence treatment for 48 and 96 h (Table 1 and Fig 2A). By comparison, the seedlings grown in normal growth conditions, the total amounts of phosphatidylcholine (PC) and phosphatidyletanolamine (PE) of 48-h and 96-h light submergence-treated, as well as phosphatidylglycerol (PG) of 48-h light submergence-treated rosettes decreased significantly (Table 1). In contrast, the total levels of phosphatidylserine (PS) and phosphatidic acid (PA) of 48-h and 96-h light submergence-treated, as well as phosphatidylinositol (PI) of 96-h light submergence-treated rosettes increased significantly. However, few differences were detected in other lipid species including digalactosyldiacylglycerol (DGDG), monogalactosyldiacylglycerol (MGDG), lysoPC, lysoPE and lysoPG in the rosettes exposed to either 48-or 96-h light submergence treatments (Table 1). With regards to the lipid compositions of different molecular species, a significant accumulation in the polyunsaturated species of PC, PE and PI, such as C34:3-PC, 36:6-PC, 34:3-PE, 34:3-PI and C36:6-PI and a decline in their saturated and mono-unsaturated species were observed at 48 h and 96 h after light submergence treatment (Fig 2A). Moreover, all the species of PA accumulated significantly, which correlated with the declined of species PC, PE or PI, except those of polyunsaturated species (34:3 and 36:6). More dramatic changes of the molecular species of PS were observed in response to light submergence. As shown in Fig 2A, the levels of unsaturated species such as 34:4-, 34:3-, 34:2-, 36:6- and 36:5-PS increased, whilst those of 36:4-, 36:3-, 36:2-, 38:4-, 38:3- and 38:2-PS declined. In particular, the VLC species of 40:3-, 40:2-, 42:3-, 42:2-, 44:3- and 44:2-PS accumulated highly upon light submergence exposure (Fig 2A). In addition, the species of plastidial lipids including 36:3-, 34:5-MGDG, 34:3- and 34:2-DGDG, as well as 34:2- and 34:1-PG decreased significantly after light submergence (Fig 2A), which may reflect the inhibition of photosynthesis by submergence. In contrast, those of polyunsaturated 34:6-MGDG/DGDG increased significantly after light submergence. Also, the contents of 36:6-MGDG/DGDG, 34:5-DGDG, and 36:5-MGDG/DGDG, 34:4-PG increased after 48-h and 96-h light submergence, respectively (Fig 2A). Since MGDG and DGDG are unique glycolipids in the photosynthetic membranes, the increases of such polyunsaturated species may be explained by the unsaturation of these galactolipids in response to submergence, which phenomenon has previously been observed during cold acclimation in *Arabidopsis* rosettes [39].

**Table 1:** Lipid profiling (% of total lipids) of 4-week-old wild-type leaves followed by light submergence treatment.

Lipid class	Control (48 h)	Control (96 h)	48 h	96 h
DGDG	15.45±0.80	14.21±1.48	16.87±0.49	13.96±0.35
MGDG	62.32±2.13	62.88±1.12	64.96±0.13	62.15±1.24
PG	6.44±0.78	7.62±0.43	**5.05±0.17** ^**b**^ *	**8.74±0.48** ^**a**^ *
PC	8.58±0.08	9.60±0.39	**6.31±0.28** ^**b**^ **	**7.44±0.27** ^**b**^ **
PE	4.21±0.41	2.89±0.16	**3.22±0.18** ^**b**^ **	**2.27±0.31** ^**b**^ *
PI	2.30±0.10	2.08±0.01	2.37±0.09	**2.98±0.15** ^**a**^ **
PS	0.27±0.03	0.27±0.02	**0.43±0.04** ^**a**^ **	**0.62±0.08** ^**a**^ **
PA	0.34±0.09	0.30±0.13	**0.66±0.11** ^**a**^ **	**2.64±0.51** ^**a**^ **
LysoPG	0.009±0.005	0.009±0.007	0.016±0.005	0.009±0.005
LysoPC	0.014±0.002	0.105±0.051	0.021±0.005	0.029±0.001
LysoPE	0.031±0.002	0.037±0.004	0.043±0.007	0.051±0.010

Values are means ±SD (% of total lipids analysed; *n* = 4). Significant differences in the samples at 48 h and 96 h followed by light submergence treatment to that of controls are in bold. Control refers to samples harvested from untreated plants.

^a^Values were higher when compared to controls in the same experiment (*P<0.05; **P<0.01).

^b^Values were lower when compared to controls in the same experiment (*P<0.05; **P<0.01).

Previous studies suggest that anoxia or hypoxia triggers production of reactive oxygen species (ROS) in plant cells [40,41], which may mediate the formation of oxidized membrane lipids non-enzymatically. To determine the effects of light submergence on the generation of oxidized lipids, we further analyzed the contents of oxidized galactolipids (MGDG and DGDG) and phospholipids (PC, PE and PG), following light submergence treatment for 48 or 96 h. The levels of arabidopsides [42], the galactolipids that conjugate to 12-oxophytodienoic acid (OPDA) and dinor-OPDA (dnOPDA), were also measured. As presented in Fig 2B, in light submergence-treated *Arabidopsis* rosettes, levels of oxidized membrane lipids as well as arabidopsides (ArA, ArB, ArC, ArD and ArE-type), were significantly higher than those of untreated controls (Fig 2B).

As compared to the dark treatment control, significant elevations in the lipid compositions of VLC-PS, PA and arabidopsides (ArA, ArB and ArE-type) were also found in 4-week-old seedlings treated with dark submergence (S3 Fig). The slight increases in species of PC, PE and PI were also observed, which were possibly due to the remarkable loss of the absolute fresh weight of plants after 24-h dark submergence exposure.

### Profiles of sphingolipids in *Arabidopsis* rosettes under submergence

**Fig 3:**
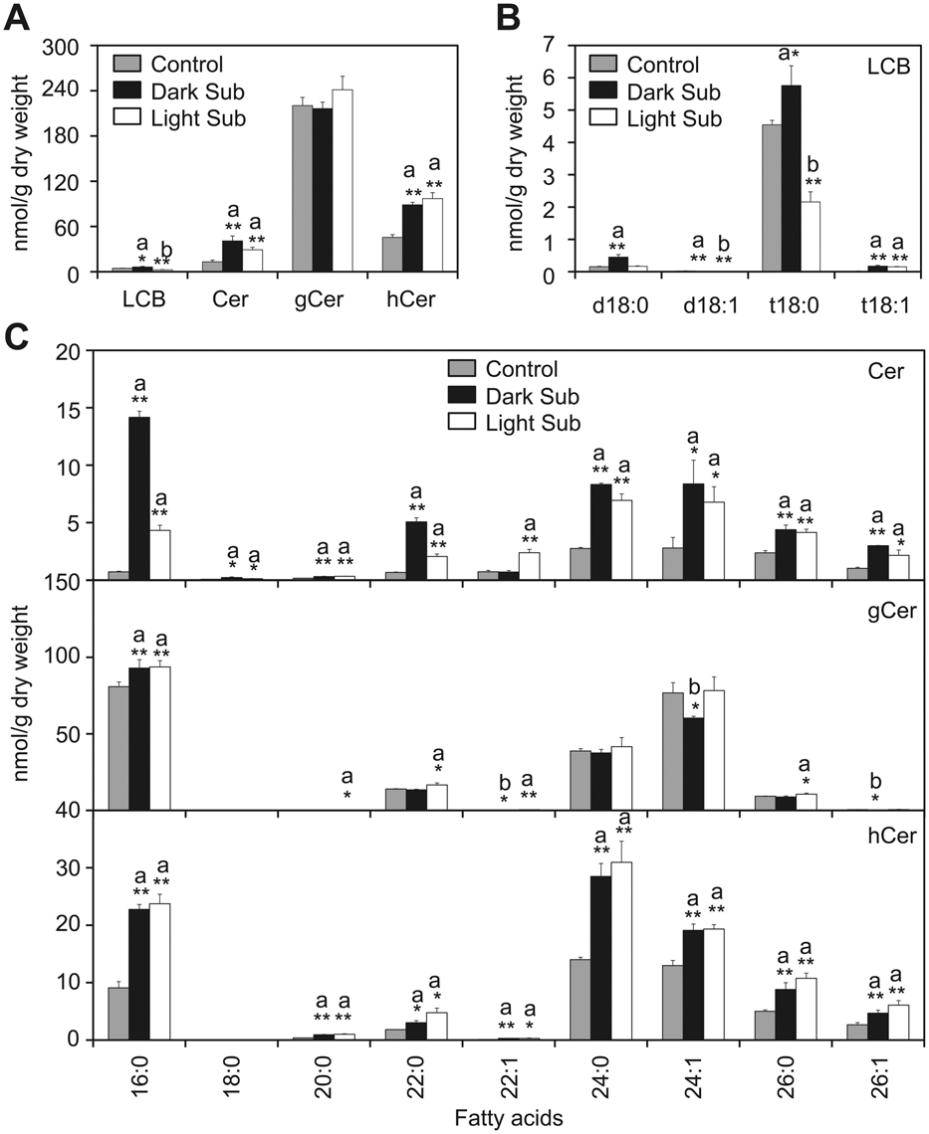
Accumulation of sphingolipids in *Arabidopsis* rosettes upon light and dark submergence exposure. (A) and (B) Total amounts of ceramides (Cer), glucosylceramide (gCer), hydroxyceramide (hCer) (A) and long-chain bases (LCB) (B) of 4-week-old *Arabidopsis* rosettes before treatment (Control) and after 24-h dark submergence or 48-h light submergence treatment (Dark Sub and Light Sub). (C) Contents of different molecular species of Cer, gCer and hCer of 4-week-old *Arabidopsis* rosettes before treatment (Control) and after 24-h dark submergence or 48-h light submergence treatment (Dark Sub and Light Sub). a, indicates value of Dark Sub or Light Sub higher than that of Control; b, indicates value of Dark Sub or Light Sub lower than that of Control (*P<0.05 or **P<0.01 by Student’s *t*-test). Values are the means ±SD (*n* = 4). Control refers to samples harvested from plants grown under the same conditions before treatment.

PS is a phospholipid enriching VLCFA, which serves as the main precursor for sphingolipid biosynthesis [43,44]. The high accumulation of VLC species of PS after light submergence treatment (Fig 2A) hinted that the remodeling of sphingolipids might be essential for plant hypoxia response. To confirm this possibility, total sphingolipids were extracted from 4-week-old *Arabidopsis* rosettes after 48-h light submergence treatment as well as untreated control, and profiled by a triple TOF LC-MS/MS system. Sphingolipids of seedlings treated with dark submergence for 24h were also analyzed and compared to those of the light submergence treatment. Results showed that both light and dark submergence treatments significantly increased the total amounts of ceramides (Cer) and hydroxyceramides (hCer), but did not affect levels of glucosylceramides (gCer) (Figs 3A and S4). The total levels of LCB and its molecular species were also significantly higher in 24-h dark submergence than for 48-h light submergence treatments or the untreated control (Fig 3A and 3B). In particular, most species of Cers (16:0, 18:0, 22:0, 24:0, 24:1, 26:0 and 26:1) were induced after 48-h light submergence by 1.7- to 6.1-fold, but were more strikingly higher in the 24-h dark submergence stress treatment, which raised them by 1.8- to 19.9-fold as compared to the control. By contrast, the level of 22:1-Cer was increased 3.3-fold by light submergence, but not by dark submergence (Fig 3C, upper panel). Additionally, the contents of most species of hCer were elicited by both submergence treatments (Fig 3C, bottom panel). Moreover, certain species of gCer were slightly affected upon either light or dark submergence exposure (Fig 3C, middle panel).

Previous studies have observed that the long-chain ceramide (C16)-containing liposomes can be delivered across cell membranes by mammalian cells [45]. To further investigate the direct link between ceramides and hypoxia response, roots of 2-week-old *Arabidopsis* seedlings were immersed in MS liquid medium containing commercially available ceramide (24:1-Cer) liposomes and whole seedlings were collected at 0, 1, 3, 6 and 12 h after treatment. Real-time PCR analysis showed that the transcripts of hypoxia-responsive genes *SUS1* and *PDC1* were induced by the 24:1-Cer treatment at various time points (6 and 12 h for *SUS1* and 3, 6 and 12 h for *PDC1*), whilst the transcript level of *ADH1* was slightly elevated by 3- and 12-h after 24:1-Cer liposome exposure (S5 Fig). However, both transcripts of *ADH1* and *PDC1* were downregulated at the early stage (1 h) after 24:1-Cer liposome treatment. In contrast, the mock treatment (dH_2_O) did not affect the transcripts of *ADH1* and *PDC1* genes in the early 24 h under normal light/dark conditions (S1 Fig). Since the gaseous hormone ethylene and its downstream response factors are known to be important for plant response to hypoxic stress [22], transcript levels of some ethylene downstream genes in the signaling cascade were also examined. Results showed that messenger RNA levels of *EIN3*, *HRE1*, *HRE2* as well as *RAP2*.*6* were upregulated, while those of *CTR1* and *EIN2* were downregulated upon 24:1-Cer liposome treatment (S5 Fig). Overall, these data indicated that ceramide is a promising functional molecule essential for the regulation of hypoxia responsive genes in *Arabidopsis*.

### Distinct responses of ceramide synthase mutants to submergence under light or dark are correlated with saturation of ceramides

**Fig 4:**
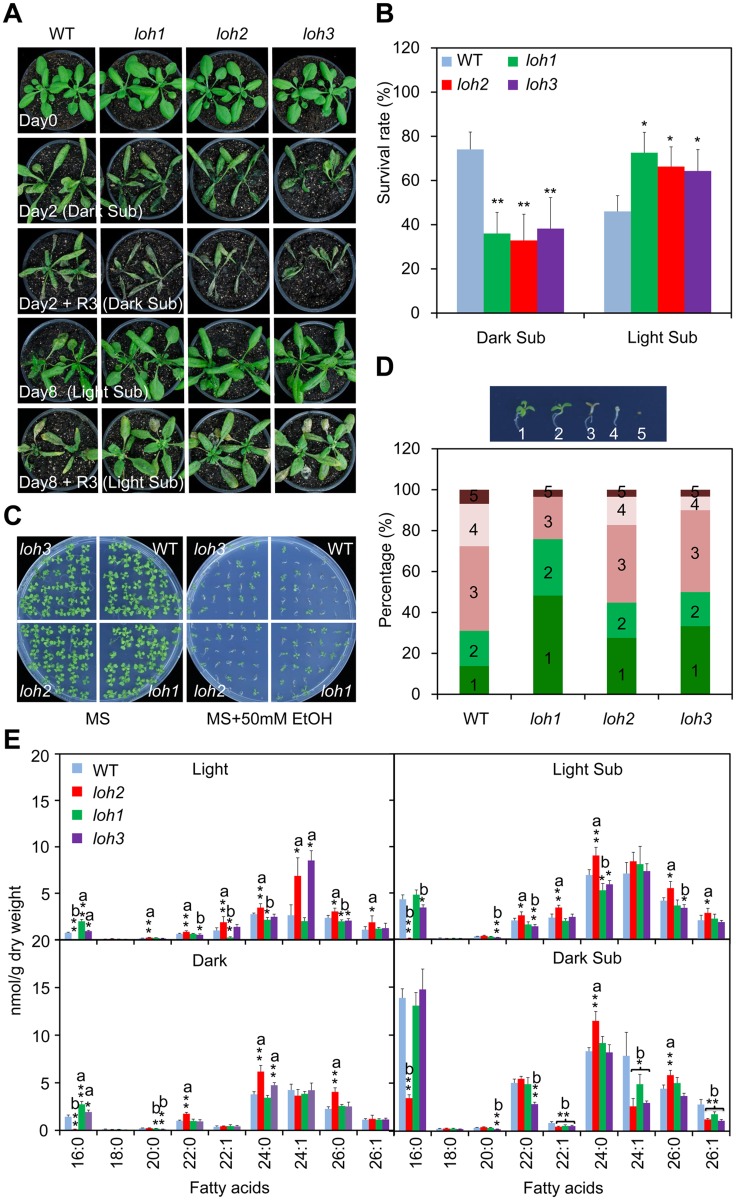
Phenotypic analysis of *loh* mutants in response to hypoxic and ethanolic treatments. (A) Images of 4-week-old wild type (WT), *loh1*, *loh2* and *loh3* mutants before treatment (Day 0), after 2-d dark submergence treatment (Day 2, Dark Sub), or 8-d light submergence treatment (Day 8, Light Sub), and followed by recovery for 3 d. The experiment has been independently repeated four times with similar results. (B) Survival rates of WT, *loh1*, *loh2* and *loh3* mutants in (A) following recovery. The survival rates were calculated based on the numbers of plants with capability to produce new leaves and continue to growth after recovery from hypoxic stress. Data are means ±SD (*n* = 60) of four independent repeats. *P<0.05 or **P<0.01 by Student’s *t*-test. (C) Images of WT, *loh1*, *loh2* and *loh3* mutants in response to 2-week ethanolic treatments. Seeds were germinated on MS medium without (MS) or with 50 μM ethanol (MS + 50 mM EtOH) for 2 weeks and images were recorded at the end of treatment. (D) Statistical frequencies of seedlings in (C). The numbers in the columns correspond to seedlings with true leaves (1), seedling with green (2) or brown (3) cotyledons, etiolated seedlings (4) and not germinated seeds (5). (E) Decrease of fatty acyl unsaturated ceramides in *loh* mutants upon dark submergence treatment in comparison to wild type. Four-week-old WT, *loh1*, *loh2* and *loh3* mutants were untreated (Light), dark-treated (Dark) and dark submergence-treated for 24 h (Dark Sub), and light submergence-treated for 48 h (Light Sub). The rosettes were harvested at the indicated times and ceramides were extracted and quantified by ESI-MS/MS. The amounts of ceramides were calculated by normalizing to the dry weights of tissues. a, indicates value of *loh* mutants higher than that of WT; b, indicates value of *loh* mutants lower than that of WT (*P<0.05 or **P<0.01 by Student’s *t*-test). Values are the means ±SD (*n* = 4).

To address the effect of ceramides on plant response to hypoxic stress, the T-DNA insertion mutants of three genes (*loh1*, *loh2* and *loh3*) encoding ceramide synthases were characterized from SALK collections [13]. PCR analysis followed by DNA sequencing localized all of the T-DNA insertions within the exons of the respective *LOH* genes (S6 Fig). The *loh1*, *loh2* and *loh3* single mutants did not exhibit visible phenotypic differences from wild type under normal growth conditions. However, all three *loh* mutants displayed enhanced sensitivity compared to the wild type, when the 4-week-old plants were dark submergence-treated for 2 d following a 3-d recovery period (Fig 4A), while a 2-d dark treatment alone did not result in obvious differences between wild type and *loh* mutants (S7 Fig). By contrast, the *loh* mutants (*loh1*, *loh2* and *loh3*) were more tolerant than wild type under light submergence conditions, for 8 d plus 3-d recovery period, as indicated by their survival rates following recovery (Fig 4A and 4B). It is noteworthy that the *loh* mutants showed significant phenotypic differences from wild type during the process of treatments (Fig 4A), indicating that the changes of submergence sensitivities in the *loh* mutants is primarily due to the interruption of plant response to hypoxia.

Under anoxia/hypoxia conditions, an early plant response is to alter the cellular metabolism from aerobic to anaerobic respiration, which thereby regenerates NAD^+^ and produces ethanol, acetaldehyde and lactate [41]. Our previous data confirmed that the final product of this reaction, ethanol, could be used to mimic hypoxic stress under certain conditions [46]. As shown in Fig 4C, when the seeds of wild type and *loh* mutants were germinated on MS solid medium supplemented with or without 50 mM ethanol (EtOH) for 2 weeks, the *loh* mutants displayed more tolerance than wild type in the presence of ethanol. Statistically, this data illustrated that the percentage of *loh* mutant seedlings with true leaves and green cotyledons were significantly higher than that of wild type (Fig 4D).

To investigate the biochemical nature for the distinct phenotypes of *loh* mutants to hypoxic stress, molecular compositions of ceramides present in their rosettes were further analyzed. Consistent with previous findings [12], the *loh2* mutant showed a dramatic reduction in ceramides with 16:0 FA, and an accumulation of VLCFAs under normal growth conditions (Light; Fig 4E). By contrast, the *loh1* and *loh3* mutants displayed slight increases in 16:0-Cer, but decreases in certain species of VLCFAs, such as 22:1-, 24:0 and 26:0-Cer in *loh1* and 20:0- and 26:0-Cer in *loh3* (Fig 4E). However, when the plants were subjected to 24-h dark submergence and 48-h light submergence treatments, more significant changes were observed in the *loh* mutants as compared to wild-type plants. As shown in Fig 4E, the mono-unsaturated VLCFA-containing ceramides including 22:1-, 24:1- and 26:1-Cer showed simultaneous decreases in the three *loh* mutants in comparison to that of wild type upon dark submergence treatment (Fig 4E). The levels of Cers with mono-unsaturated-VLCFA were not significantly altered between light submergence-treated wild type and *loh* mutants, whereas the levels of saturated species such as 16:0-Cer in *loh2* and *loh3* mutants, 24:0-Cer in *loh1*, and 20:0-, 22:0-, 24:0- and 26:0-Cer in *loh3*, were significantly reduced as compared to wild type (Fig 4E).

**Fig 5:**
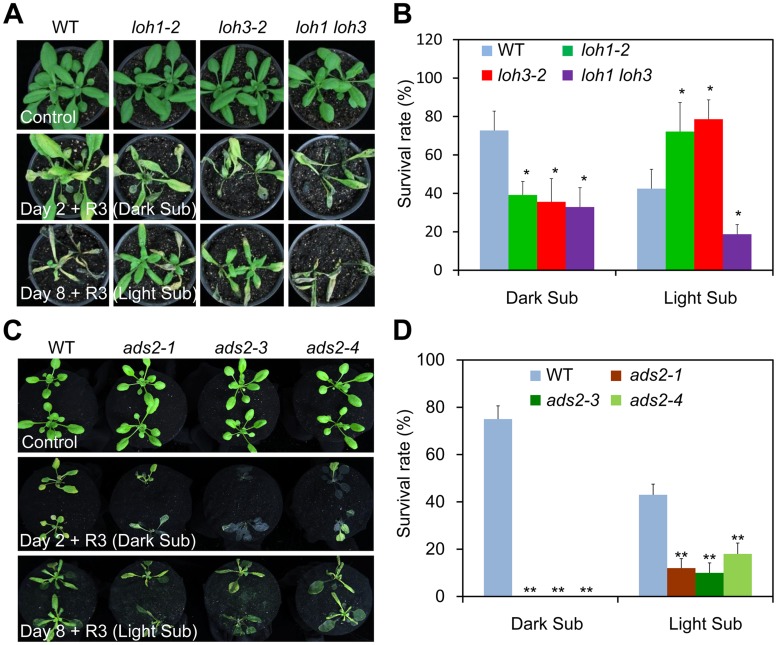
The *loh1 loh3* knockdown double mutant and *ads2* mutants displayed attenuated tolerance to both dark and light submergence. (A) The response of *loh1-2*, *loh3-2* and *loh1 loh3* mutants to hypoxia. Four-week-old wild type (WT), *loh1-2*, *loh3-2*, as well as *loh1 loh3* were untreated (Control) or dark submergence-treated for 2 d (Day 2 + R3, Dark Sub), or light submergence-treated for 8 d (Day 8 + R3, Light Sub), followed by recovery for 3 d. The experiment has been independently repeated with similar results. (B) Survival rates of WT, *loh1-2*, *loh3-2* and *loh1 loh3* in (A) following recovery. (C) The response of *ads2-1*, *ads2-3* and *ads2-4* mutants to hypoxia. Three-week-old WT, *ads2-1*, *ads2-3* and *ads2-4* plants were untreated (Control) or dark submergence-treated for 2 d (Day 2 + R3, Dark Sub), or light submergence-treated for 8 d (Day 8+ R3, Light Sub), followed by recovery for 3 d. The experiment has been independently repeated with similar results. (D) Survival rates of WT, *ads2-1*, *ads2-3* and *ads2-4* in (C) following recovery. The survival rates in (B) and (D) were calculated based on the numbers of plants with capability to produce new leaves and continue to growth after recovery from hypoxic stress. Data are means ±SD (*n* = 20). *P<0.05, **P<0.01 by Student’s *t*-test.

To further confirm the different responses of the *loh* mutants to light and dark submergence stresses, two additional *loh* mutants, *loh1-2* and *loh3-2*, as well as the knockdown double mutant *loh1-1 loh3-1* [12], were exposed to either light or dark submergence conditions. As shown in Fig 5, the *loh1-2* and *loh3-2* mutants responded similarly, *i*.*e*., enhanced sensitivity under dark submergence and greater tolerance to light submergence treatments than wild type. However, the *loh1-1 loh3-1* double mutant displayed attenuated tolerance to both dark and light submergence stresses in comparison with wild type (Fig 5A). In agreement with the phenotypic observations, the survival rates of *loh1-2* and *loh3-2* mutant plants were higher under light submergence but lower under dark submergence conditions, than those of wild type (Fig 5B). In contrast, the survival rates of *loh1-1 loh3-1* double mutant under both dark and light submergences were significantly lower than wild type (Fig 5B).

Given the remarkable reduction of VLC species of Cers in *loh1-1 loh3-1* double mutant [12], these data suggest that levels of Cers with unsaturated or saturated VLC species may contribute to the differential phenotypes of *loh* mutants to dark and light submergence stresses. We further tested this hypothesis using the *ads2* (*ads2-1*, *ads2-3* and *ads2-4*) mutants, which exhibited a specific deficiency in the VLC unsaturated Cers in *Arabidopsis* [18], in comparison to the wild type plants. As expected, all the *ads2* mutants were more sensitive to both dark and light submergence treatments than wild type (Fig 5C and 5D), indicating the unsaturation of VLC species plays an essential role in controlling submergence tolerance.

### Ceramides interact with CTR1 protein kinase *in vitro*

**Fig 6:**
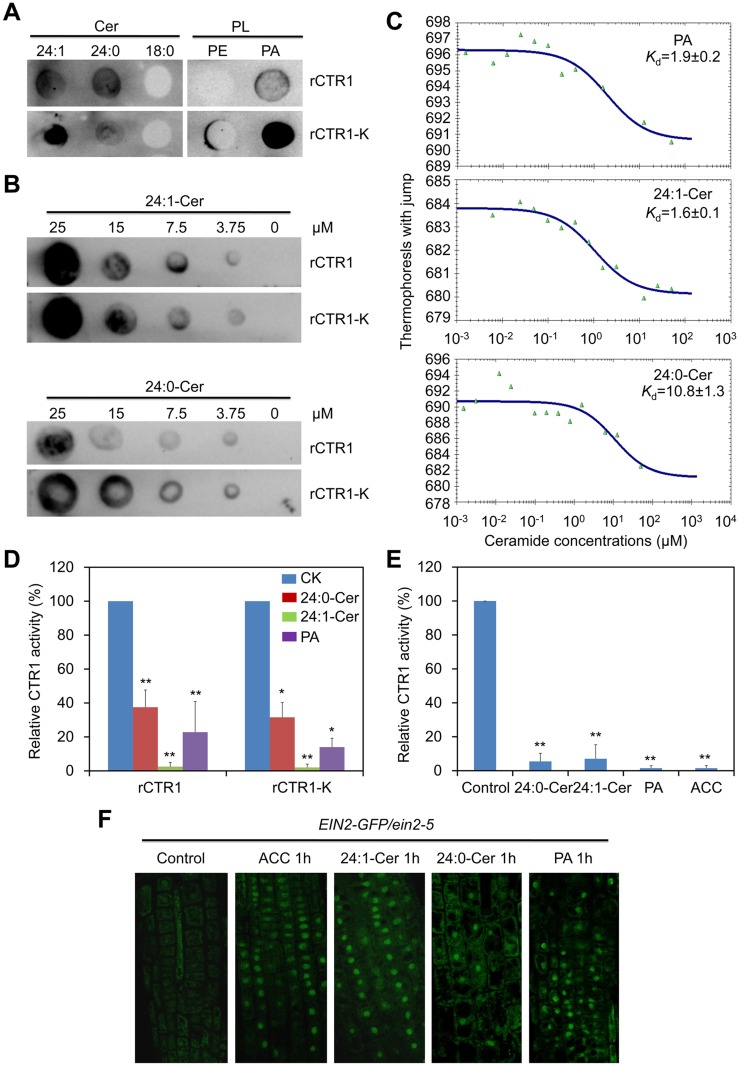
Ceramides interact with the kinase domain of CTR1 protein *in vitro*. (A) Recombinant rCTR1 and CTR1 deletion mutant with kinase alone (rCTR1-K) binds ceramides/phospholipids on filters. Fifty micromole concentrations of various ceramides (24:1-Cer, 24:0-Cer and 18:0-Cer) and phospholipids (PE and PA) were spotted onto nitrocellulose and incubated with 1 μg/mL of either purified rCTR1/rCTR1-K protein. The binding was detected by immunoblotting using anti-GST antibodies and anti-His antibodies, respectively. (B) Binding of the rCTR1 and rCTR1-K to 24:1- and 24:0-Cer on filters. Various concentrations (0, 3.75, 7.5, 15 and 25 μM) of 24:1- and 24:0-Cer spotted onto nitrocellulose. (C) MST analysis of the interaction between rCTR1 and liposomes of PA, 24:1-Cer as well as 24:0-Cer. (D) Measurement of CTR1 kinase activity *in vitro*. The purified proteins (rCTR1 and rCTR1-K) were pre-incubated without or with 1 nM 24:0-ceramide, 24:1-ceramide and PA liposomes and subsequently used for kinase activity assay. The relative TR-FRET signals were calculated based on the fluorescence emission ratio at 665/620 nm and presented by subtracting the signals of negative controls purely containing the substrate without ligands. The signals of rCTR1 or rCTR1-K pre-incubated without liposomes were set as 100% and the relative CTR1 activities in the proteins pre-incubated with 24:0-ceramide, 24:1-ceramide and PA liposomes were calculated accordingly. The experiment has been repeated and data are means ±SD (*n* = 10). *P<0.05, **P<0.01 by Student’s *t*-test. (E) Measurement of CTR1 kinase activity *in vivo*. The 10-d-old *Arabidopsis* seedlings (WT and *ctr1-1*) were non-treated or treated with 10 μM ACC, 50 μM 24:0-ceramide, 24:1-ceramide and PA liposomes for 1 h and their total proteins were extracted for kinase assay. The relative TR-FRET signal was calculated based on the fluorescence emission ratio at 665/620 nm and presented by subtracting the background signal in the *ctr1-1* mutant from WT. The signals of non-treated proteins were set as 100% and the relative CTR1 activities in the proteins treated with 24:0-ceramide, 24:1-ceramide and PA liposomes as well as ACC were calculated accordingly. The experiment has been repeated and data are means ±SD (*n* = 10). **P<0.01 by Student’s *t*-test. (F) Activation of EIN2-GFP translocation from the ER to the nucleus by ceramides. Roots of 7-d-old EIN2-GFP/*ein2-5* seedlings were used for detection of GFP fluorescence at 1 h after treatment with either 10 μM ACC, 50 μM 24:0-Cer, 24:1-Cer and PA liposomes.

Given ceramides can specifically interact with the protein kinase Raf-1 in mammals [31,32], we considered the potential interaction between ceramides and the *Arabidopsis* Raf kinase CTR1 (S8 Fig); the latter was suggested to bind PA through the C-terminal kinase domain [47]. To test this hypothesis, the recombinant proteins consisting of CTR1 full-length protein (rCTR1) as well as the C-terminal kinase domain alone (rCTR1-K) were expressed and purified from *Escherichia coli*. Membrane dot binding assays indicated that both rCTR1 and rCTR1-K proteins bound PA, but not PE, confirming previous reports using PA beads [47]. To rule out the possibility that binding of rCTR1 and rCTR1-K proteins to lipids is due to non-specific hydrophobic association, a wider range of membrane lipid class, including PA, PC, PE, PG, PI, PS, MGDG and DGDG was used to test lipid binding (S9A Fig). The result again verified the binding specificity between rCTR1/rCTR1-K proteins and PA (S9B Fig). We further observed that both recombinant proteins bound 24:0- and 24:1-Cer but not 18:0-Cer by the same binding approach (Fig 6A). When various concentrations of 24:0- and 24:1-Cer were applied, both rCTR1 and rCTR1-K proteins bound 24:1-Cer better than 24:0-Cer (Fig 6B). By using an independent microscale thermophoresis (MST) analysis, our data showed that the rCTR1-24:1-Cer interaction was comparable to that of rCTR1-PA binding, with a dissociation constant (*K*_d_) of 1.6 and 1.9 μM, respectively (Fig 6C and Table 2). Furthermore, we found that both rCTR1 and rCTR1-K bound 24:1-Cer with higher affinities than that of 24:0-Cer, as reflected by the *K*_d_ values (Fig 6C). These results revealed that CTR1 preferably binds unsaturated VLC ceramides via the C-terminal kinase domain *in vitro*.

**Table 2:** Dissociation constant (*K*_d_) for the binding of rCTR1 and rCTR1-K to liposomes of PA, 24:1-Cer, 24:0-Cer and 18:0-Cer.

Proteins	Dissociation Constants (*K*_d_)			
	PA	24:1-Cer	24:0-Cer	18:0-Cer
rCTR1	1.9±0.2	1.6±0.1	10.8±1.3	50.6±5.2
rCTR1-K	1.3±0.4	1.0±0.1	4.3±1.3	49.3±7.0

### Ceramides modulate the kinase activity of CTR1, the ER-to-nucleus translocation of EIN2-GFP and the stability of EIN3-GFP *in vivo*

To investigate the biological significance of the interactions between CTR1 and unsaturated VLC ceramides, the kinase activity of CTR1 upon treatments with 24:0-Cer, 24:1-Cer and PA liposomes was measured in both *in vitro* and *in vivo* assays using the TR-FRET technique. As presented in Fig 6D, when the purified proteins were pre-incubated with 1 nM liposomes containing 24:1-Cer and PA, both rCTR1 and rCTR1-K proteins exhibited decreased kinase activities in comparison to that of an untreated control. Consistent with its lower binding affinity to rCTR1 and rCTR1-K proteins (Fig 6B and 6C), 24:0-Cer liposome inhibited CTR1 activity with lesser effect than 24:1-Cer and PA liposomes (Fig 6D). Moreover, the *in vivo* kinase activity was measured using total proteins extracted from wild-type and the *ctr1-1* seedlings treated with 50 μM 24:0-Cer, 24:1-Cer and PA liposomes. As a positive control, the seedlings were also treated with 10 μM ACC. By subtracting the background signal from the *ctr1-1* mutant, *Arabidopsis* wild-type seedlings treated with ACC as well as 24:0-Cer, 24:1-Cer and PA liposomes showed significant lower CTR1 specific kinase activities than the control treatment (Fig 6E), indicating that applications of 24:0-Cer, 24:1-Cer and PA liposomes inhibit the CTR1 kinase activity significantly.

To further address the effects of the CTR1-Cer interaction on the downstream signaling of CTR1 protein, the EIN2-GFP reporter was used to determine whether ceramide application could interfere with the processing and ER-to-nucleus translocation of EIN2 protein, whose activities are directly regulated by CTR1 in ethylene signaling pathway [48–50]. To this end, 7-d-old seedlings of EIN2-GFP grown on MS medium were placed in MS liquid medium supplemented with 10 μM ACC, 50 μM 24:0-Cer or 24:1-Cer liposomes for 1 h and root tip cells were subsequently observed by confocal microscopy. As presented in Fig 6F, the nuclear accumulation of EIN2-GFP fluorescence was evident upon 24:0-Cer, 24:1-Cer and PA liposome treatments, with significantly higher numbers of fluorescent nuclei in the root cells treated with 24:1-Cer liposomes. As controls, EIN2-GFP was observed to quickly move from the ER membrane to the nucleus after 1-h ACC stimulation but no such movement was detected during the mock treatment (Control; Fig 6F).

**Fig 7:**
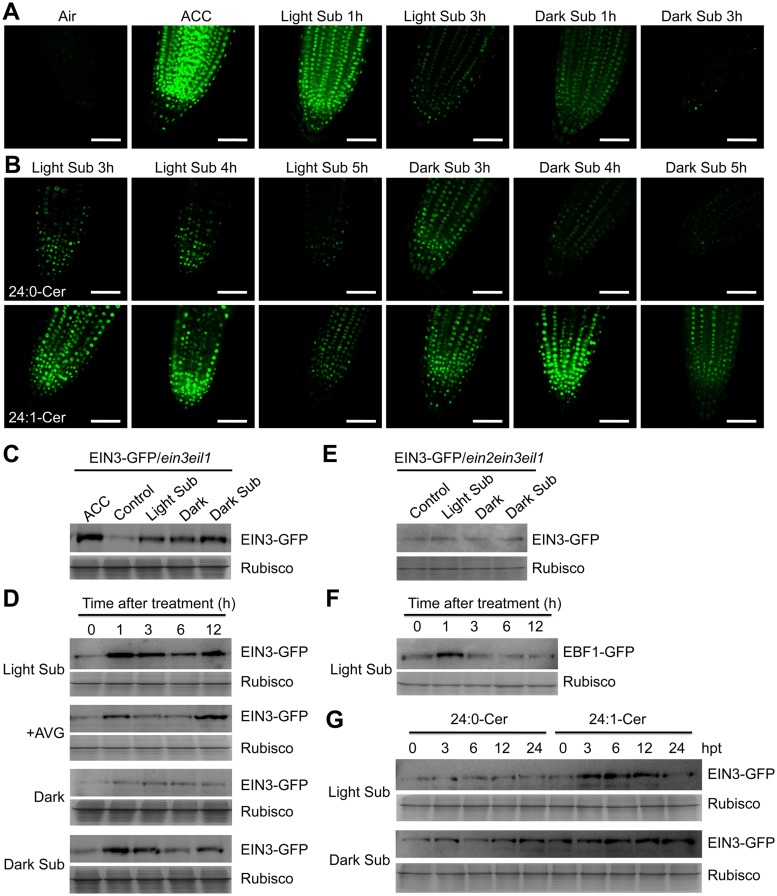
Stabilization of EIN3-GFP protein by ceramides and hypoxia. (A) EIN3-GFP fusion protein is stabilized by light submergence (Light Sub) or dark submergence (Dark Sub) treatment. Roots of 7-d-old *35S*:*EIN3-GFP* transgenic lines in *ein3 eil1* background (EIN3-GFP/*ein3 eil1*) were sampled for detecting GFP fluorescence after light submergence or dark submergence treatment for 1 h and 3 h. Treatment with ACC (10 μM) for 1 h was set as a positive control. Experiments were carried out in triplicate with similar results. Bars = 50 mm. (B) Accumulation of EIN3-GFP fusion protein by exogenous application of ceramides under light submergence or dark submergence conditions. Roots of 7-d-old EIN3-GFP/*ein3 eil1* seedlings were used for detection of GFP fluorescence at 3, 4 and 5 h after light submergence or dark submergence treatments with 50 μM 24:0-Cer or 24:1-Cer liposome. Experiments were carried out in triplicate with similar results. Bars = 50 mm. (C) Immunoblot analysis showing the levels of EIN3-GFP fusion protein upon light submergence and dark submergence treatments. EIN3-GFP/*ein3 eil1* seedlings (7-d-old) treated with light submergence or dark submergence were collected at 3 h after treatment. The untreated samples (Control) and treated with ACC (ACC) and darkness (Dark) for 3 h were used as controls. (D) Immunoblot analysis showing the levels of EIN3-GFP protein upon light submergence, AVG (2 μM), Dark, dark submergence treatments during various time points. EIN3-GFP/*ein3 eil1* seedlings were treated and harvested at 0, 1, 3, 6 and 12 h after treatment. (E) Immunoblot analysis showing the levels of EIN3-GFP fusion protein upon light submergence and dark submergence treatments in *ein2 ein3 eil1* triple mutant background (EIN3-GFP/*ein2 ein3 eil1*). EIN3-GFP/*ein2 ein3 eil1* seedlings (7-d-old) were treated with light submergence or dark submergence for 6 h. The untreated samples (Control) and treated with darkness (Dark) for 6 h were used as controls. (F) Degradation of EBF1-GFP fusion protein upon light submergence exposure. *35S*:*EBF1-GFP* seedlings (7-d-old) were light submergence-treated and harvested at 0, 1, 3, 6 and 12 h after treatment. (G) Differential effects of 24:0 and 24:1 ceramides on the light submergence- and dark submergence-induced accumulations of EIN3-GFP fusion protein. EIN3-GFP/*ein3 eil1* seedlings (7-d-old) were light submergence or dark submergence-treated with 50 μM 24:0-Cer or 24:1-Cer liposome and samples were harvested at 0, 3, 6, 12 and 24 h after treatment. (C) to (G) Anti-GFP antibodies were applied for protein blotting analysis. Coomassie blue-stained total proteins (Rubisco) are shown on lower panels for each treatment to indicate the amount of protein loaded per lane.

Based on the above results, we hypothesize that light submergence-triggered production of 24:1-Cer might be a signal for activation of the ethylene pathway through interaction with the kinase domain of CTR1 and inhibition of its activity. As a confirmation, the EIN3-GFP fusion transgenic lines in the *ein3 eil1* background (whose stabilization is used as a marker to indicate the downstream ethylene response) [51] were used to explore the effect of ceramides on ethylene signaling. Seven-d-old EIN3-GFP/*ein3 eil1* seedlings were treated with ACC, light submergence, or dark submergence, and the stability of EIN3-GFP fluorescence was monitored by confocal microscopy. Similar to the application of ACC, which was reported to stabilize EIN3-GFP protein [51], both light and dark submergence stresses effectively induced the accumulation of EIN3-GFP fusion protein in root tip cells (Fig 7A). EIN3-GFP fusion rapidly accumulated and peaked at 1 h after light or dark submergence treatment, and then gradually decreased from 1 to 3 h. Furthermore, the accumulation of EIN3-GFP fusion was significantly greater in light submergence-treated cells than those of dark submergence-treated cells (Fig 7A). To further explore the effects of ceramides on the light submergence- and dark submergence-induced accumulations of EIN3-GFP, 50 μM 24:0-Cer liposomes or 50 μM 24:1-Cer liposomes were added to the light submergence- or dark submergence-exposed EIN3-GFP/*ein3 eil1* lines and subsequently incubated for 3, 4, or 5 h. Results showed that application of 24:1-Cer liposomes under either light or dark submergence condition culminated in enhanced accumulation of EIN3-GFP fusion in the nucleus, in contrast to the singular treatments of either 24:0-Cer liposomes, light submergence, or dark submergence (Fig 7B). We have shown that under normal conditions, the *loh2* and *loh3* mutants had enriched endogenous 24:1-Cer than 24:0-Cer in comparison to those of the *loh1* mutant and wild type (Fig 4E).

To verify the results from confocal microscopy analyses, immunoblotting was performed to detect the EIN3-GFP protein levels in the various treatments. The data showed that EIN3-GFP fusions were induced by light and dark submergence treatments in the *ein3 eil1* double mutant background; the former displayed a much higher level upon treatment (Fig 7C and 7D). Moreover, when the EIN3-GFP fusion was introduced into the *ein2-5 ein3 eil1* triple mutant background, the light submergence- or dark submergence-inducible stabilization of EIN3-GFP protein was blocked (Fig 7E), suggesting that EIN2 is required for hypoxia-inducible EIN3-GFP accumulation. In contrast, EBF1-GFP fusion proteins were degraded upon light submergence-treatment (Fig 7F). When liposomes of 24:0-Cer and 24:1-Cer were applied to light submergence- or dark submergence-treated EIN3-GFP/*ein3 eil1* lines for 0, 3, 6, 12, or 24 h, both treatments supplied with 24:1-Cer liposomes accumulated significantly higher levels of EIN3-GFP protein than the 24:0-Cer liposome application (Fig 7G).

**Fig 8:**
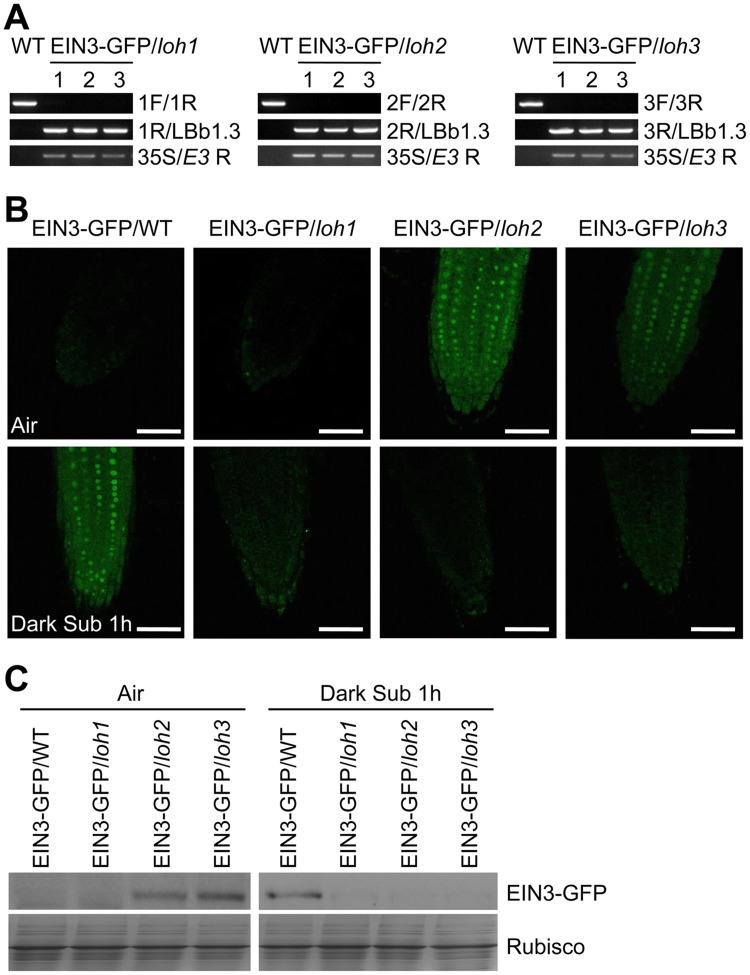
Stabilization of EIN3-GFP protein by endogenous ceramides catalyzed by ceramide synthases in the *loh* mutants. (A) Genotyping of the EIN3-GFP/*loh1*, EIN3-GFP/*loh2* and EIN3-GFP/*loh3* plants by PCR. The *loh1*, *loh2* and *loh3* mutants were crossed to the EIN3-GFP/WT line, and named EIN3-GFP/*loh1*, EIN3-GFP/*loh2* and EIN3-GFP/*loh3*, respectively. Genomic DNAs extracted from wild type (WT), EIN3-GFP/*loh1*, EIN3-GFP/*loh2* and EIN3-GFP/*loh3* were amplified using the primer pairs indicated on the right. (B) EIN3-GFP signal in the root tip cells of 7-d-old EIN3-GFP/WT, EIN3-GFP/*loh1*, EIN3-GFP/*loh2* and EIN3-GFP/*loh3* lines under normal growth conditions (Air) and after dark submergence treatment for 1 h (Dark Sub 1h). Experiments have been repeated with similar results. Bars = 50 mm. (C) Immunoblot analysis showing the levels of EIN3-GFP protein in EIN3-GFP/WT, EIN3-GFP/*loh1*, EIN3-GFP/*loh2* and EIN3-GFP/*loh3* lines before and after submergence. One-week-old seedlings were untreated or treated by light submergence for 1 h. Anti-GFP antibodies were applied for blotting. Coomassie blue-stained total proteins (Rubisco) are shown on lower panels to indicate the amount of protein loaded per lane.

To test the *in vivo* effects of C24:1-Cer on ethylene signalling, the EIN3-GFP fusion was expressed in *loh1*, *loh2* and *loh3* backgrounds (Fig 8A) and the stability of EIN3-GFP were compared among them. In comparison with the EIN3-GFP/WT line, the EIN3-GFP protein was clearly enhanced in both *loh2* and *loh3* backgrounds in the root tip cells under normal growth conditions (Fig 8B and 8C). Also, weak EIN3-GFP fluorescence was detected in the *EIN3-GFP/loh1* line, possibly due to the increase of long-chain ceramides in the *loh1* mutant (Fig 4E). We further found that the EIN3-GFP stabilities were significantly decreased by dark submergence for 1 h in all of the EIN3-GFP/*loh1*, EIN3-GFP/*loh2* and EIN3-GFP/*loh3* mutant lines, in comparison with the inducible accumulation of EIN3-GFP protein in the EIN3-GFP/WT line (Fig 8B and 8C). These results are consistent with the ceramide profiling data (Fig 4E) and suggest that the cellular levels of unsaturated ceramides (such as C24:1-Cer) is primarily associated with the stability of EIN3 protein *in vivo*.

Together, these results imply that the unsaturated ceramides appear to regulate hypoxia response by modulating the kinase activity of CTR1 in the ethylene signaling pathway.

### The hypersensitivity of *loh* mutants to dark submergence stress depends on CTR1

**Fig 9:**
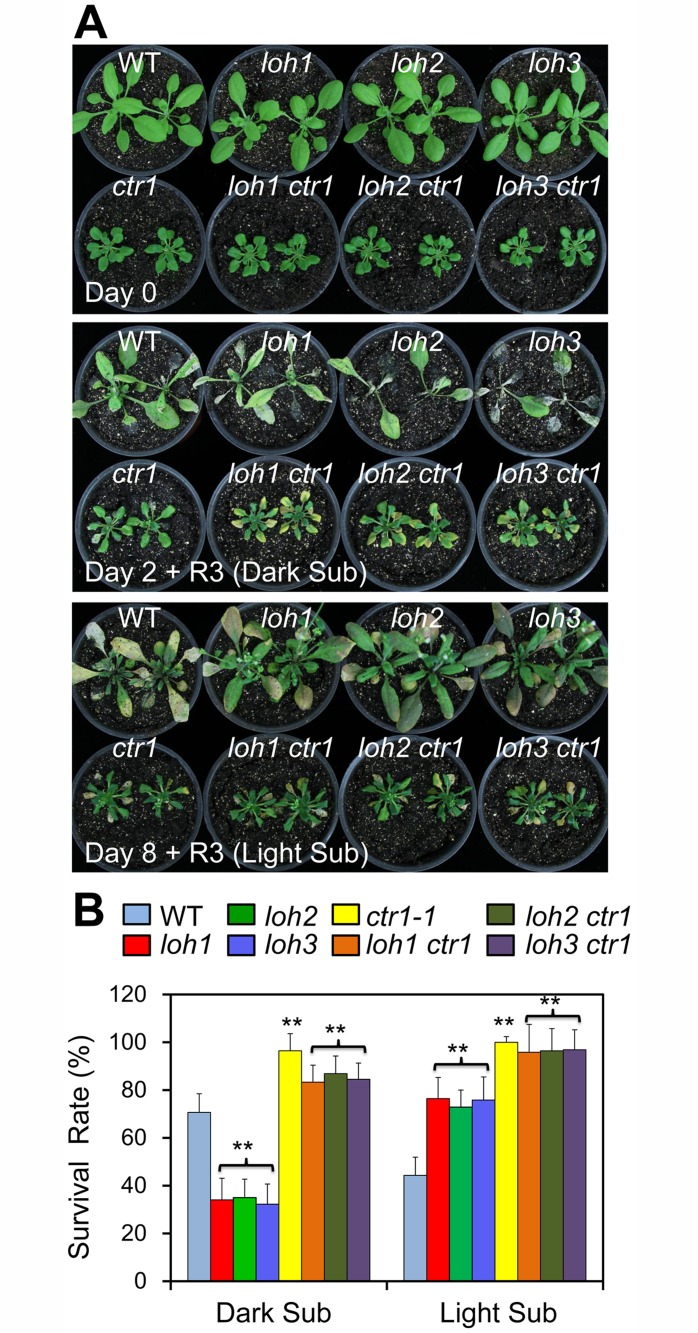
The hypoxia-sensitive phenotypes of *loh* mutants under dark submergence were rescued by *ctr1-1*. (A) Phenotypic analysis of wild type (WT), *loh* mutants, *ctr1-1* mutant and *loh ctr1* double mutants in response to dark submergence or light submergence treatment. Photos were taken 3 d after recovery from 2 d of dark submergence treatment, or 8 d of light submergence treatment. (B) Survival rates of WT, *loh* mutants, *ctr1-1* mutant and *loh ctr1* double mutants in (A) following recovery. The survival rates were calculated based on the numbers of plants with capability to produce new leaves and continue to growth after recovery from hypoxic stress. The experiments have been repeated 3 times and similar results were obtained. Data are means±SD (*n* = 20). **P<0.01 by Student’s *t*-test.

To understand the genetic link between ceramide and ethylene signaling, the *loh1*, *loh2* and *loh3* mutants were crossed to the constitutive triple response mutant *ctr1-1* [52], and the *loh1 ctr1*, *loh2 ctr1* and *loh3 ctr1* double mutants were characterized. As shown in Fig 9A, all the double mutants displayed dwarfish phenotypes, resembling that of the *ctr1-1* single mutant. When the plants were treated with dark submergence, single mutant phenotypes of *loh1*, *loh2* and *loh3* were rescued by the *ctr1-1* mutant, as indicated by the improved performance of the *loh1 ctr1*, *loh2 ctr1* and *loh3 ctr1* double mutants in comparison with the corresponding *loh* single mutants under hypoxia (Fig 9A and 9B). These results confirmed that ethylene signaling is likely a downstream event regulated by ceramides.

## Discussion

In plants, cellular membrane integrity and fluidity are largely determined by the lipid composition and the extent of desaturation, which further influences bilayer permeability, ATPase activity, and membrane-associated transportation [3]. It is generally recognized that the unsaturation of membrane lipids is an important factor in plant response to various environmental stresses such as chilling, freezing, heating, salinity and drought [53–58]. For example, unsaturated fatty acids accumulate in *Arabidopsis* rosettes upon exposure to chilling or freezing temperatures, whereas unsaturated species decrease markedly as the growth temperature increases to levels of heat stress [54,55]. Morever, two independent studies suggest that in *Arabidopsis*, the levels of unsaturated fatty acids are significantly increased in response to shoot-specific hypoxia or in crown galls under hypoxia/drought conditions, which processes are controlled by the phosphate-responsive transcription factor PHR1 and desaturases SAD6/FAD3, respectively [59,60]. In addition, our recent findings suggest that the acyl-CoA-binding protein ACBP3 is involved in plant response to hypoxia by interacting with very-long-chain (VLC) acyl-CoA esters and modulating VLC-fatty acid metabolism in *Arabidopsis* [46]. These results demonstrate that the dynamic maintenance of lipid metabolism and proper functionality of cellular membranes are vital for plant responses to various hypoxic stresses. In this investigation, we present further evidence to show the dynamic profiles of lipid composition in *Arabidopsis* in response to hypoxic stress, which was mimicked by completely submerging *Arabidopsis* seedlings under light or dark conditions. Our data indicate that hypoxia-induced lipid changes in *Arabidopsis* rosettes resemble lipid profiles of tissues upon chilling or freezing treatments [39], including a significant decrease in the levels of galactolipids and phospholipids, but elevations of unsaturated glycerolipid species, PA, as well as oxidized membrane lipids. Specifically, hypoxia activated the accumulation of VLCFA-enriched PS as well as its derivative ceramides in *Arabidopsis* rosettes (Figs 2 and 3). Therefore, our findings indicate that the unsaturation of VLC-ceramides is likely to be a protective mechanism that promotes tolerance to hypoxic stress in *Arabidopsis*.

The importance of ceramides in hypoxia responses has recently been demonstrated in *C*. *elegans* and mammalian cells [28,30,61]. Our results presented here further extend the significance of hypoxia-inducible ceramides in plants, and reveal that ceramides are conserved signal molecules among eukaryotic species essential for regulation of hypoxic/anoxic adaptation. Earlier studies primarily focused on the overall ceramide species of the hypoxia-sensitive mutants under normal conditions rather than specific correlation analysis between the phenotypes and ceramide profiles upon hypoxia, and therefore multiple mechanistic understandings have been proposed. Devlin et al. [30] showed that hypoxia induces a rapid increase of all species of dihydroceramide (DHCs) in mammals, which returns to basal levels in a short time once the hypoxic stress recedes. The activity of DHC desaturase (DEGS) responsible for *de novo* DHC synthesis is oxygen-dependent, and overexpression of DEGS improves cell proliferation under hypoxia [30,62]. These findings suggest that the DEGS-triggered desaturation of DHCs is a potential oxygen sensor for synthesis of ceramides and it balances different bioactive ceramide species during hypoxic conditions. Similar to the existence of two types of ceramide synthases in *Arabidopsis*, there are two genes *HYL-1* and *HYL-2* encoding ceramide synthase in *C*. *elegans*; they show distinct roles in the synthesis of C24-26 and C20-22 ceramides *in vivo* [28,29]. Since deletions of *HYL-1* and *HYL-2* genes lead to attenuated and enhanced anoxia sensitivities, respectively, the C20-22 ceramides generated by *HYL-2* appeared to be protective against anoxic stress [28,29]. In *Arabidopsis*, the *loh1*, *loh2* and *loh3* mutants exhibited indistinguishable enhanced tolerances following light hypoxia treatments (Fig 4), suggesting that the different acyl-chain lengths of VLC-ceramides are not essential for plant responses to hypoxia. However, depletion of light by treating plants under conditions of dark hypoxia, all three *loh* mutants were hypersensitive to hypoxic stress (Fig 4). Consistently, the desaturated species of VLC-ceramides declined in the *loh* mutants, indicating the involvement of a light-dependent fatty acyl desaturase in hypoxia-induced desaturation of VLC-ceramides. Moreover, the VLC-ceramides in *Arabidopsis* are produced by the redundant function of LOH1 and LOH3, whose knockout double mutant is embryo lethal [12]. By utilizing a leaky double mutant *loh1-1 loh3-1*, in which the *LOH1* and *LOH3* were downregulated and the levels of VLC-ceramides were substantially reduced [12], we observed that the light-induced tolerant phenotypes were compromised in the *loh1-1 loh3-1* line (Fig 5). Therefore, our findings identified a novel VLC fatty acyl desaturation-dependent rather than the typically acyl chain length-related mechanism during hypoxia adaptation in *Arabidopsis*. In fact, several components of lipid metabolism including an acetyl-CoA carboxylase ACCase, two acyl-CoA-binding proteins ACBP4 and ACBP5, and a fatty acyl desaturase FAD7, are transcriptionally modulated by light/dark cycling [2]. Analysis of transgenic lines expressing *Arabidopsis FAD7* promoter fusion with β–glucuronidase reporter has shown that expression of *FAD7* is activated by light and suppressed by constant darkness [63], suggesting that lipid desaturation is tightly regulated by light to satisfy cellular lipid demands in plant cells. A recent investigation has uncovered a role for ADS2, an *Arabidopsis* acyl-CoA desaturase-like enzyme, in predominantly catalyzing the mono-desaturation of C24- and C26-ceramides [18]. Given that ADS2 is involved in lipid remodeling during establishment of cold acclimation which is a light-dependent process [64,65], and is also required for hypoxic tolerance under both dark and light submergence conditions (Fig 5), we therefore suggest that ADS2 is an essential light-activated desaturase for hypoxia-triggered desaturation of VLC-ceramides in *Arabidopsis*.

One contradictory result arising from this study is that the unsaturated species of VLC-ceramides were not upregulated in the light submergence-tolerant *loh* mutants. Instead, significant decreases of saturated species of long-chain ceramides (16:0) and some VLC-ceramides (20:0, 22:0 and 24:0) were observed in the light submergence-treated *loh* mutants (Fig 4E). It is well-known that the structural variation of ceramides causes differential physiological functions in plant cells [44]. Previous studies have clearly demonstrated that hydroxylation and phosphorylation of LCB and ceramides are important for their capability to induce cell death [17, 66–68]. Although the contribution of fatty acyl desaturation in modulating cell death in plant cells is still unclear, it is conceivable that the enhanced resistance of *loh* mutants under light submergence conditions is possibly due to the decrease of saturated ceramides, the accumulation of which may induce hypoxic injury and promote cell death in plant cells.

In higher plants, the gaseous hormone ethylene is a central signaling molecule in regulation of diverse hypoxia responses such as early hypoxia sensing, hypoxia-responsive gene regulation, and development of survival strategies [22]. In this report, we present several lines of evidence to show that ceramides regulate hypoxic tolerance by modulating ethylene signaling. Firstly, exogenous application of ceramides activated the transcription of hypoxia- and ethylene-responsive factors HRE1 and HRE2 [22, 38] (S5 Fig). Secondly, the C24 species of VLC-ceramides interacted with the kinase domain of recombinant CTR1 protein with high affinity *in vitro* (Fig 6). Thirdly, the unsaturated ceramides stimulated the ER-to-nucleus translocation of EIN2-GFP and stabilized EIN3-GFP fusion protein *in vivo* (Figs 6, 7 and 8). Finally, the enhanced sensitivities of *loh* mutants to dark submergence were rescued by introduction of the *ctr1-1* mutation that constitutively induces ethylene responses (Fig 9).

In mammals, several targets of ceramides have been identified including protein kinases Raf-1 and PKCζ [31,69–71] and protein phosphatases [72,73]. Ceramides, together with PA and PS, are well-known to serve as lipid cofactors involved in activation of Raf-1 and its subsequent signal transduction cascade [32,74]. CTR1 is a Raf-1-like protein kinase active downstream of ethylene receptors, and a key negative regulator in ethylene signaling [47]. In the absence of ethylene, CTR1 directly interacts with ethylene receptors and represses ethylene responses by maintaining the downstream elicitor EIN2 at the ER [49,75]. In contrast, the presence of ethylene inhibits CTR1 activity and subsequently stimulates the phosphorylation-dependent processing and ER-to-nucleus movement of EIN2 protein, resulting in accumulation of EIN3/EIL1 and activating ethylene responses [48–50]. Our data further showed that the binding of 24:0-Cer or 24:1-Cer to CTR1 caused a reduction of its kinase activity in both *in vitro* and *in vivo* assays (Fig 6), suggesting that although both protein kinases Raf-1 and CTR1 bind ceramides, their underling mechanisms appear to be different. Unlike the positive activation of Raf-1 kinase by ceramides in mammalian cells [31], our findings indicate that ceramides, at least their VLC species, are likely to inhibit the CTR1 activity and CTR1-mediated signaling in *Arabidopsis*. By analysis of transgenic lines expressing EIN2-GFP, we observed that the processing and nuclear translocation of this protein was enhanced upon treatment with unsaturated VLC-ceramides (Fig 6), supporting the theory that CTR1 activity is suppressed by ceramides. Consistently, the ceramide-induced accumulation of EIN3-GFP (Fig 7) and enhanced expression of downstream transcription factors *HRE1*, *HRE2* and *RAP2*.*6* further confirms this hypothesis (S5 Fig). In support, a recent investigation showed that the recombinant CTR1 protein binds to PA and suppresses the activity of CTR1 *in vitro* [47]. Nevertheless, the biological significance of CTR1-PA interaction *in vivo* remains to be determined. Thus, further identification of the lipid-binding domain and specific binding sites in CTR1 is needed to deepen our understanding of the importance of lipid cofactors in the regulation of CTR1 function.

In conclusion, we described a novel mechanism of unsaturation of VLC ceramides in protecting *Arabidopsis* from hypoxia-induced damages. Based on the present data that in response to hypoxia, the unsaturated VLC-ceramides bind to CTR1 and activate the subsequent downstream ethylene responses, we propose that unsaturated VLC-ceramides may function in modulating ethylene signaling and hypoxia response. Furthermore, the balance between the saturated and unsaturated species of VLC ceramides may govern the cell death and cell survival responses upon plant exposure to a hypoxic environment.

## Materials and Methods

### Plant materials and growth conditions

The *Arabidopsis* T-DNA insertion mutants of *LOH1*, *LOH2* and *LOH3* genes were identified from the SALK collections (http://signal.salk.edu) with locus names of *loh1* (SALK_069253), *loh2* (SALK_018608C), *loh3* (SALK_150849), which were previously described by Ternes et al. [13]. The characterization of *loh1-2* and *loh3-2* mutants as well as *loh1-1 loh3-1* double mutant, and generation of transgenic lines expressing *35S*:*EIN3-GFP* in *ein3 eil1* and *ein2 ein3 eil1* backgrounds, as well as *35S*:*EBF1-GFP* transgenic lines in wild-type (Col-0) background have been reported [12,51]. The knockout mutants of *ads2-1* (SALK_079963C), *ads2-3* (CS817934) and *ads2-4* (CS873338) were characterized following previous descriptions [18, 64]. The *ctr1-1* mutant [52] was obtained from The *Arabidopsis* Information Resource (TAIR; http://www.arabidopsis.org).

For germination assays, *Arabidopsis* seeds were surface-sterilized with 20% bleach containing 0.1% Tween-20 for 20 min, and then washed 5 times with sterile water. Seeds were sown on MS medium, followed by cold treatment in the dark for 3 d. After germination for 7 d, seedlings were transplanted to soil and grown in a plant growth room with a 16-h-light (125 μmol m^-2^ s^-1^)/8-h-dark cycle at 22°C.

### Hypoxic and ethanolic treatments

Hypoxic treatment was carried out following the method of Licausi et al. [24] with minor modifications. Briefly, 3- or 4-week-old plants were submerged at depths of 5–10 cm beneath the water surface for 8 d under light conditions (60 μmol m^-2^ s^-1^; light submergence; Light Sub) or for 2 d under constant darkness (dark submergence; Dark Sub). Plant samples were collected or photographed at the indicated times. Dry weights and survival rates were recorded after 3-d recovery. The survival rates were calculated based on the numbers of plants with capability to produce new leaves and continue to grow after recovery from hypoxic stress.

For the ethanolic treatment, simultaneous harvested seeds were sterilized and sown on MS solid medium with or without 50 mM ethanol. Following cold treatment for 2 d, the seeds were germinated under a 16-h-light/8-h-dark cycle at 22°C. Seedlings were scored and photographed at 2 weeks after germination.

### Microarray analysis

Microarray analysis was performed as described previously [76]. Wild-type (Col-0; 4-week-old) plants were light submergence-treated and rosettes were harvested at 0 and 48 h after treatment. Each sample included three biological replicates, and each replicate collected from three independent plants. Total RNA was extracted using the RNeasy Plant Mini kit (Qiagen) according to the manufacturer’s instructions. Labeling, hybridization, scanning, and detection on the ATH1 *Arabidopsis* chips (Affymetrix), as well as raw data collection using the Affymetrix Gene Chip software MAS 5.0 were carried out as previously described [76]. The data were deposited on the Gene Expression Omnibus (GEO) database under accession number GSE59719. In addition, Affymetrix CEL files of short-term anoxia (6 h) in GSE2133 [77], hypoxia (2 and 9 h) in GSE9719 [78], dark submergence treatment in GSE24077 [36] were also applied for analysis together with light submergence (48 h) data. The genes involved in lipid biosynthesis and metabolism were identified according to the pathways described in Li-Beisson et al. [7] and Nakamura et al. [79]. GeneSpring 12.6 was used to identify differential expression genes with the criterion of 1.5-fold or more change and *P* < 0.05 cutoffs for the subsequent analysis. R language was used for all calculations and plots.

### Measurements of polar membrane lipids and sphingolipids

For membrane lipid analysis, the total plant lipids were extracted following the method of Welti et al. [39]. The profiles of membrane lipids were determined by automated electrospray ionization–tandem mass spectrometry as previously described [42,80]. The data of polar membrane lipids described in this work were acquired from the Kansas Lipidomics Research Center Analytical Laboratory. Instrument acquisition and lipidomics method development was supported by National Science Foundation (EPS 0236913, MCB 0920663, DBI 0521587, DBI1228622), Kansas Technology Enterprise Corporation, K-IDeA Networks of Biomedical Research Excellence (INBRE) of National Institute of Health (P20GM103418), and Kansas State University.

Extraction of sphingolipids was carried out according to method IV in Markham et al. [81] using 100 mg tissue samples. Extracts were dried under nitrogen, then dissolved in 1 mL of methanol and analyzed on a triple TOF 5600 MS/MS system (AB SCIEX, Canada). Separations were accomplished on an Agilent Eclipse XDB C8 column (50×2.1 mm, 1.8 μm). The column heater temperature was maintained at 40°C. The mobile phases were composed of 100% methanol and 1 mM ammonium formate with 0.2% formic acid and the flow rate kept at 0.3 mL/min. The sample volume injected was set at 10 μL. The conditions of MS/MS detector were as follows: temperature 450°C; curtain gas 30 psi; flow rate 10 L/min; ion spray voltage 5,000 V. Quantification was performed by normalizing the peak areas to the internal standards and response factors as previously described [82,83].

### Ceramide treatment, RNA extraction and real-time PCR analysis

Liposomes were prepared according to Hu et al. [84]. For ceramide treatment, 2-week-old wild type (Col-0) seedlings were floated on MS liquid medium containing 0.1 mM ceramide liposomes (24:1-Cer). The samples were collected at 0, 1, 3, 6 and 12 h after treatment.

Total RNA was isolated and real-time PCR (qPCR) was analyzed as described previously [76]. Gene-specific primers used for qPCR analysis are listed in S6 Table.

### Purification of recombinant proteins and lipid-protein interaction assays

The constructs of rCTR1 and rCTR1-K fusion were obtained by amplifying the *CTR1* cDNA coding region fragment or CTR1 kinase domain alone using primer pairs XS1483/XS1484 and XS1181/XS1182 (S6 Table), respectively, by RT-PCR. The confirmed PCR fragments were inserted into GST-tagged expression vector pGEX-6P-1 and (His)_6_-tagged expression vector pRSET A, respectively. The constructs were transformed into *Escherichia coli* BL21 (DE3), and the recombinant proteins were expressed and purified following the manufacturer’s instructions (Invitrogen).

Binding of rCTR1 and rCTR1-K to ceramides and phospholipids on filters was carried out as previously described [42,85,86]. For MST analysis, the three recombinant proteins were first labeled with the Monolith NT Protein Labeling Kit RED. Labeled proteins were used at a concentration of 10 nM in 1× phosphate buffer saline (pH 7.6) containing 0.05% Tween-20. The concentration of various liposomes of ceramides and PA (Avanti) ranged from 1.5 nM to 50 μM. An optimized buffer (50 mM Tris-HCl pH 7.4, 150 mM NaCl, 10 mM MgCl_2_, 0.05% Tween-20) was prepared for incubation of proteins and liposomes for 5 min. The combined solution of labeled proteins and liposomes were transferred into standard treated capillaries and MST was measured on a Nano Temper Monolith NT.115 (20% LED power; 50% laser power).

### Measurement of protein kinase activity

The kinase activities of rCTR1 and rCTR1-K proteins (20 ng) or *Arabidopsis* total proteins (3 μg) from 10-d-old seedlings were measured using the STK Substrate 2-biotin in HTRF KinEASE kit according to the manufacturer’s instructions. In brief, assays were conducted in low volume, white 384-well plates (Corning Life Sciences, MA), with a 20 μl assay volume containing 100 μM ATP, 1 μM STK Substrate 2-biotin. For recombinant proteins, liposomes such as 24:0-ceramide, 24:1-ceramide and PA (1 nM, Avanti) were pre-incubated with protein at 4°C for 30 min. For total proteins, *Arabidopsis* seedlings were non-treated or treated with 10 μM ACC, 50 μM 24:0-ceramide, 24:1-ceramide and PA liposomes and the total proteins were extracted using kinase buffer containing 250 mM (pH 7.0) HEPES, 0.1% NaN_3_, 0.05% BSA, 0.5 mM Orthovanadate, 2 mM DTT and 10 mM MgCl_2_. The kinase reaction was carried out following incubation at room temperature for 1 h. The reaction was stopped with buffered EDTA followed by the fluorescent development for 1 h at room temperature. The resulting specific TR-FRET signal was detected by a TECAN detection system (Infinite M1000) and calculated based on the fluorescence emission ratio at 665/620 nm.

### Laser scanning confocal microscopy

The EIN3-GFP/*loh1*, EIN3-GFP/*loh2* and EIN3-GFP/*loh3* lines were generated by genetic crossing *35S*:*EIN3-GFP* line to *loh1*, *loh2* and *loh3* mutants, respectively. The GFP fluorescence in the primary roots of 7-d-old *35S*:*EIN2-GFP*, *35S*:*EIN3-GFP*, EIN3-GFP/*loh1*, EIN3-GFP/*loh2* and EIN3-GFP/*loh3* lines was observed at the indicated time points after light submergence- or dark submergence-treated in liquid MS medium, or by application of 50 μM 24:0-ceramide and 24:1-ceramide liposomes (Avanti). The treatments with 10 μM ACC and 2 μM AVG were set as controls. A TCS-SP5 laser scanning confocal microscope (Leica) was used for analysis the stabilization of EIN3-GFP fusion protein. GFP fluorescence was excited at 488 nm, filtered through a primary dichroic (UV/488/543), a secondary dichroic of 545 nm and subsequently through 500–600 nm emission filters to the photomultiplier tube (PMT) detector.

### Protein extraction and immunoblot analysis

Total proteins were extracted by grinding samples in liquid nitrogen followed by adding ice-cold extraction buffer (50 mM sodium phosphate, pH 7.0, 200 mM NaCl, 10 mM MgCl_2_, 0.2% *β*-mercaptoethanol and 10% glycerol) supplemented with the protease inhibitor cocktail (Roche). Extracts were placed on ice for 30 min, and then centrifuged at 11,000 *g* for 30 min to collect the supernatant for electrophoresis.

For immunoblot analysis, total proteins were subjected to SDS-PAGE and electrophoretically transferred to Hybond-C membrane (Amersham). The anti-GFP (Roche; 1:3,000) antibodies were used in the protein blotting analysis.

### Generation of *loh1 ctr1*, *loh2 ctr1* and *loh3 ctr1*double mutants

The *loh1 ctr1*, *loh2 ctr1* and *loh3 ctr1* double mutant combinations were generated by genetic crossing parental single homozygous lines of *loh1*, *loh2* and *loh3* [13] to *ctr1-1* [52]. The T-DNA insertions of *loh1*, *loh2* and *loh3* were identified by screening all of the F_2_ population using gene-specific primers and paired with a T-DNA-specific primer LBb1.3 (S6 Table). The seedlings displaying constitutive triple-response phenotypes were deemed to be homozygous for *ctr1-1* [52].

### Accession numbers

The Arabidopsis Genome Initiative numbers for genes mentioned in this article are as follows: *LOH1* (At3g25540), *LOH2* (At3g19260), *LOH3* (At1g13580), *ADS2* (At2g31360), *CTR1* (At5g03730), *EIN2* (At5g03280), *EIN3* (At3g20770), *ADH1* (At1g77120), *PDC1* (At4g33070), *SUS1* (At1g01040), *HRE1* (At1g72360), *HRE2* (At2g47520), *RAP2*.*6* (At1g43160), *RAP2*.*12* (At1g53910), *HB1* (At2g16060), *HUP09* (At5g10040) and *LBD41* (At3g02550).

## Supporting Information

S1 Fig Transcripts of hypoxia responsive genes after light submergence treatment. Total RNA was isolated from 4-week-old WT seedlings exposed to light submergence treatment. The samples were collected at 0, 12, 24, 48 and 72 h after treatment and the relative expression levels of hypoxia responsive genes (*ADH1*, *PDC1*, *HUP09*, *HB1*, *LBD41* and *RAP2*.*12*) were determined by real-time PCR analysis. Expression levels of each time point were normalized to both 0 h and *ACTIN2*. The experiments were repeated and similar results were obtained. Values represent means ±SD (*n* = 3). *P<0.05 or **P<0.01 by Student’s *t*-test.
(TIF)

S2 Fig Differentially expressed genes involved in fatty acid biosynthesis (A), fatty acid degradation (B), glycerolipid metabolism (C and D), cuticular lipid metabolism (E and F) and sphingolipids metabolism (G) under hypoxic stress. For (D) and (F), the hierarchical cluster analysis was applied to the 38 DEGs in cuticular lipid metabolism and 26 DEGs glycerolipid metabolism in the selected anoxia, hypoxia and submergence stresses (Anoxia 6h, Hypoxia 2h, Hypoxia 9h, Dark Sub 7h, Dark Sub 24h and Light Sub 48h). The data of anoxia, hypoxia and dark submergence treatments were exported from Gene Expression Omnibus database choosing 6-h anoxia in GSE2133 [77], 2-h and 9-h hypoxia in GSE9719 [78], and 7-h and 24-h dark submergence in GSE24077 [36]. The transcriptional profiles of relative gene expression values (log2 scale of microarray value) were analyzed using the heatmap command of the R language. Red and blue colors represent upregulated and downregulated genes, respectively.
(TIF)

S3 Fig Compositions of phospholipids and oxidized lipids in *Arabidopsis* rosettes upon dark submergence exposure. (A) Contents of different molecular species of phospholipids (PC, PE, PI, PS and PA) in 4-week-old wild-type *Arabidopsis* after dark treatment (Dark 24 h) and dark submergence treatment (Dark Sub 24 h). (B) Amounts of oxidized membrane lipid species (MGDG-O, DGDG-O, PG-O, PC-O and PE-O) as well as complex arabidopsides (ArA, ArB, ArC, ArD and ArE-type) in 4-week-old wild-type *Arabidopsis* after dark treatment (Dark 24 h) and dark submergence treatment (Dark Sub 24 h). Values represent means ±SD (*n* = 4). *P<0.05 or **P<0.01 by Student’s *t*-test.
(TIF)

S4 Fig Sphingolipids profiles of *Arabidopsis* rosettes upon light and dark submergence exposure. Contents of ceramides (Cer, gCer and hCer) of 4-week-old *Arabidopsis* rosettes before treatment (Control) and after 24-h dark submergence (Dark Sub) or 48-h light submergence (Light Sub) treatment. Data was normalized according to hydroxylation of LCBs (A) and saturation extends of LCBs (B) in ceramides. Values represent means ±SD (*n* = 4). *P<0.05 or **P<0.01 by Student’s *t*-test.
(TIF)

S5 Fig Expression patterns of hypoxia responsive genes in response to ceramide application. Total RNA was isolated from 2-week-old wild type (WT) seedlings treated with ceramides by floating on MS liquid medium containing 0.1 mM ceramide liposomes (Cer 24:1). The samples were collected at 0, 1, 3, 6 and 12 h after treatment and the relative expression levels of hypoxia responsive genes (*ADH1*, *PDC1*, *SUS1*, *CTR1*, *EIN2*, *EIN3*, *HRE1*, *HRE2* and *RAP2*.*6*) were determined by real-time PCR analysis. Expression levels of each time point were normalized to both 0 h and *ACTIN2*. The experiments have been repeated and similar results were obtained. Values represent means ±SD (*n* = 3).*P<0.05 or **P<0.01 by Student’s *t*-test.
(TIF)

S6 Fig Molecular characterization of T-DNA insertional mutants in *LOH1*, *LOH2* and *LOH3* genes. (A) T-DNA insertion sites of the knockout mutants within *LOH1* (AT3G25440), *LOH2* (AT3G19260) and *LOH3* (AT1G13580) genes. Primers designed for genotyping are indicated. White and black boxes indicate UTRs and exons, respectively. Lines between the black boxes indicate introns. (B) Genotyping of the *loh1*, *loh2* and *loh3* mutants by PCR. Genomic DNA extracted from wild type (WT), *loh1*, *loh2* and l*oh3* mutants was amplified using the primer pairs indicated on the right. (C) RT-PCR (electrophoretogram) and qRT-PCR (column chart) analyses showing the knockout or knockdown transcriptions of *LOH* genes in the *loh1*, *loh2* and *loh3* mutants, respectively. Total RNAs isolated from WT, *loh1*, *loh2* and *loh3* mutants were employed for RT-PCR or qRT-PCR analyses.
(TIF)

S7 Fig Control plants of wild type (WT) and *loh* mutants by 2-d dark treatment. Four-week-old WT, *loh1*, *loh2* and *loh3* mutants were placed under constant dark condition for 2 d, and followed by recovery for 3 d. Few phenotypic difference between WT and *loh* mutants were observed. The experiment has been independently repeated four times with similar results.
(TIF)

S8 Fig Alignment of kinase domains from CTR1 with human Raf-1. Sequence alignment showing the conserved lipid binding sites K(R)601, R(K)602 and R604 in the kinase domains of *Arabidopsis* CTR1 and human Raf-1 proteins.
(TIF)

S9 Fig Interaction of rCTR1 and rCTR1-K proteins with the membrane lipids *in vitro*. Twenty-five micromole concentrations of various lipids (PA, PG, PE, PC, PI, PS, MGDG and DGDG) were spotted onto nitrocellulose (A) and incubated with 1 μg/mL of either purified rCTR1 or rCTR1-K protein. The interaction between proteins and lipids was detected by immunoblotting using anti-GST antibodies (for rCTR1) or anti-His antibodies (for rCTR1-K) (B).
(TIF)

S1 Table List of DEGs in *Arabidopsis* rosettes upon 48-h light submergence treatment. (XLSX)

S2 Table List of DEGs in fatty acid biosynthesis and degradation. (XLSX)

S3 Table List of DEGs in the sphingolipid metabolism pathway. (XLSX)

S4 Table List of DEGs in the wax metabolism pathway. (XLSX)

S5 Table List of DEGs in the glycerolipid metabolism pathway. (XLSX)

S6 Table Sequences of primers used in this study. (DOC)
