# Supplementary figures and images for: Unsaturation of Very-Long-Chain Ceramides Protects Plant from Hypoxia-Induced Damages by Modulating Ethylene Signaling in Arabidopsis
Source: PLoS Genet. 2015 Mar 30;11(3):e1005143. doi: 10.1371/journal.pgen.1005143 (PMC4379176; doi:10.1371/journal.pgen.1005143)

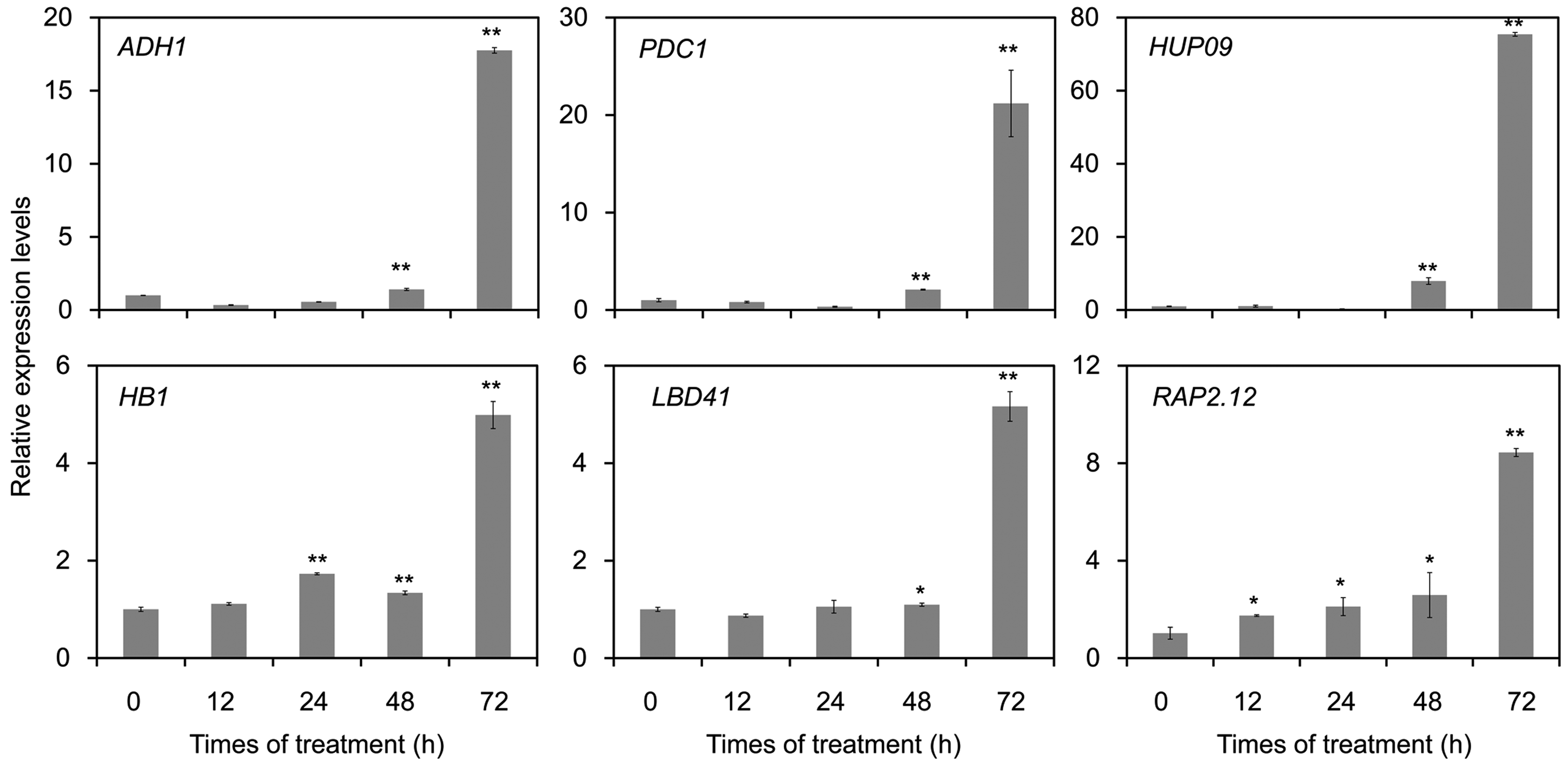

Supplement: S1 Fig — Total RNA was isolated from 4-week-old WT seedlings exposed to light submergence treatment. The samples were collected at 0, 12, 24, 48 and 72 h after treatment and the relative expression levels of hypoxia responsive genes (ADH1, PDC1, HUP09, HB1, LBD41 and RAP2.12) were determined by real-time PCR analysis. Expression levels of each time point were normalized to both 0 h and ACTIN2. The experiments were repeated and similar results were obtained. Values represent means ±SD (n = 3). *P<0.05 or **P<0.01 by Student’s t-test. (TIF) [file pgen.1005143.s001.tif]

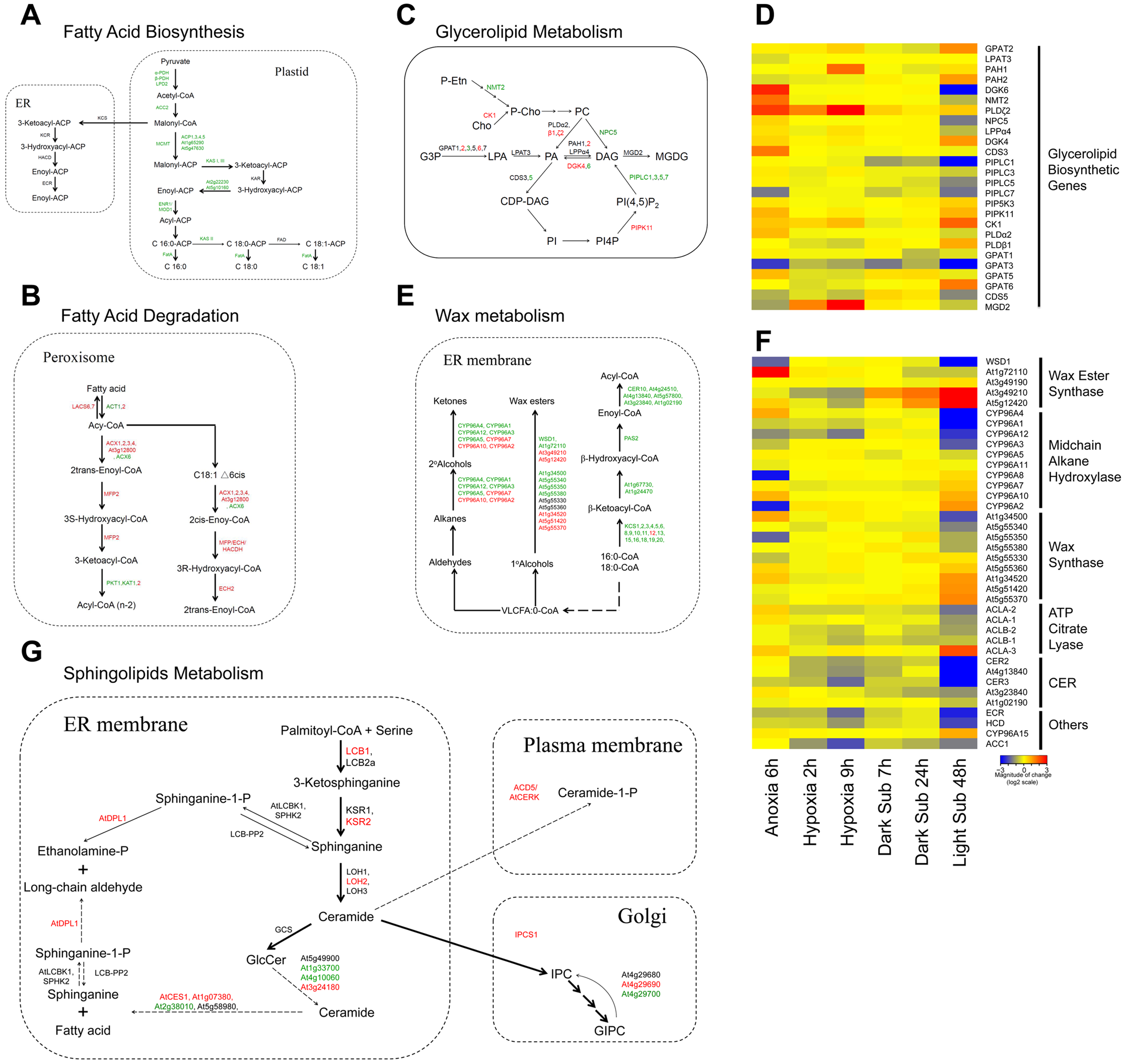

Supplement: S2 Fig — For (D) and (F), the hierarchical cluster analysis was applied to the 38 DEGs in cuticular lipid metabolism and 26 DEGs glycerolipid metabolism in the selected anoxia, hypoxia and submergence stresses (Anoxia 6h, Hypoxia 2h, Hypoxia 9h, Dark Sub 7h, Dark Sub 24h and Light Sub 48h). The data of anoxia, hypoxia and dark submergence treatments were exported from Gene Expression Omnibus database choosing 6-h anoxia in GSE2133 [77], 2-h and 9-h hypoxia in GSE9719 [78], and 7-h and 24-h dark submergence in GSE24077 [36]. The transcriptional profiles of relative gene expression values (log2 scale of microarray value) were analyzed using the heatmap command of the R language. Red and blue colors represent upregulated and downregulated genes, respectively. (TIF) [file pgen.1005143.s002.tif]

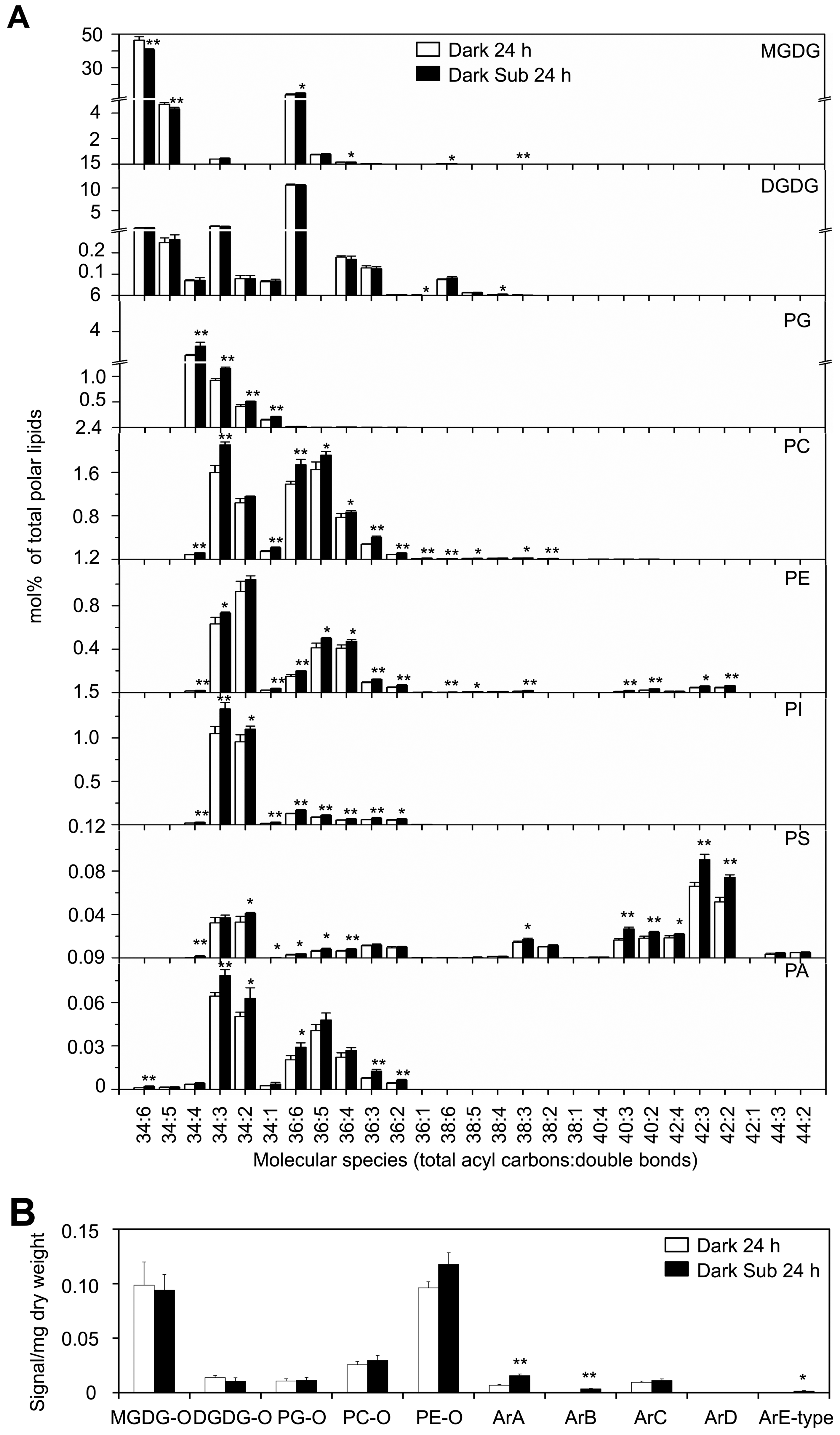

Supplement: S3 Fig — (A) Contents of different molecular species of phospholipids (PC, PE, PI, PS and PA) in 4-week-old wild-type Arabidopsis after dark treatment (Dark 24 h) and dark submergence treatment (Dark Sub 24 h). (B) Amounts of oxidized membrane lipid species (MGDG-O, DGDG-O, PG-O, PC-O and PE-O) as well as complex arabidopsides (ArA, ArB, ArC, ArD and ArE-type) in 4-week-old wild-type Arabidopsis after dark treatment (Dark 24 h) and dark submergence treatment (Dark Sub 24 h). Values represent means ±SD (n = 4). *P<0.05 or **P<0.01 by Student’s t-test. (TIF) [file pgen.1005143.s003.tif]

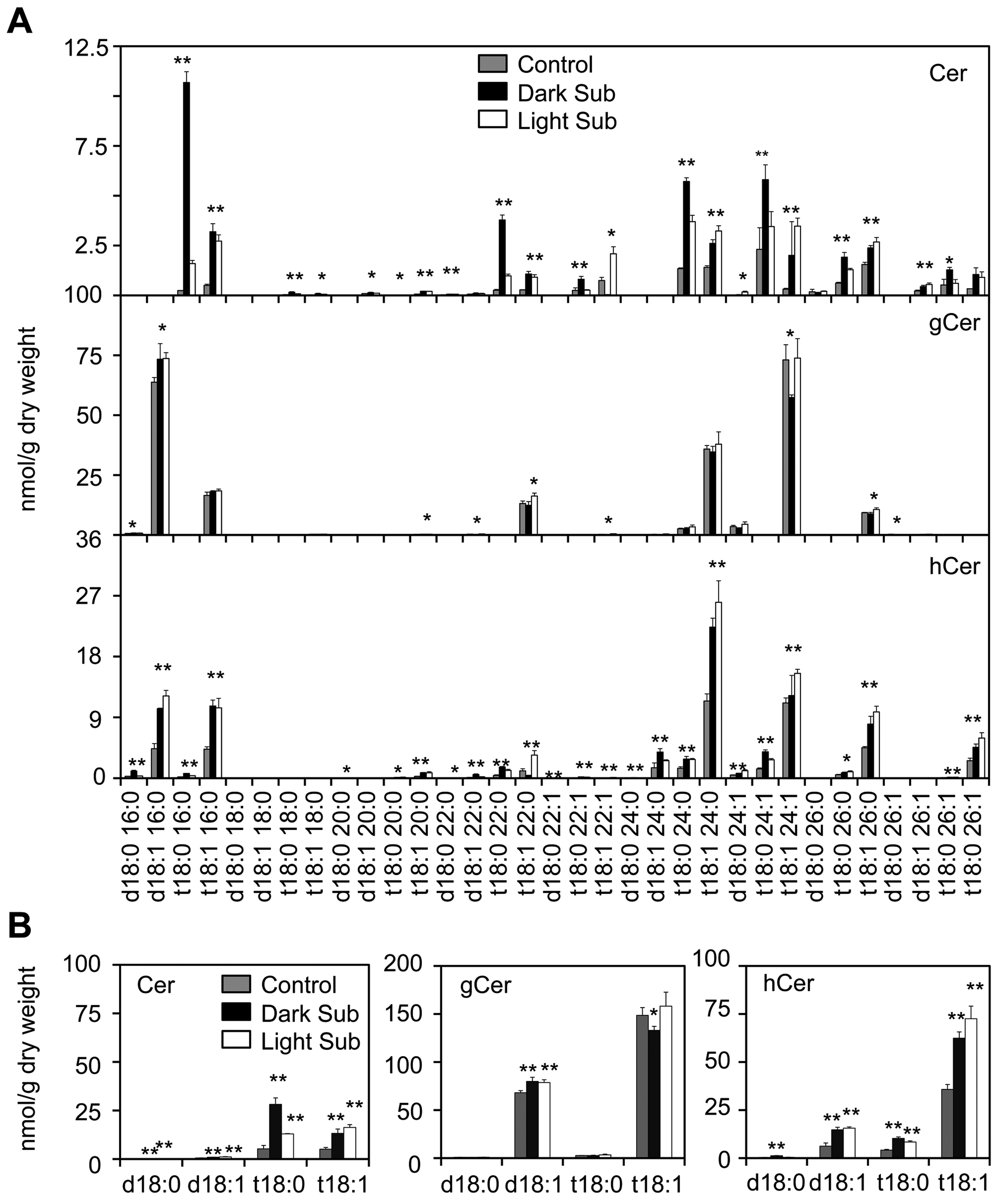

Supplement: S4 Fig — Contents of ceramides (Cer, gCer and hCer) of 4-week-old Arabidopsis rosettes before treatment (Control) and after 24-h dark submergence (Dark Sub) or 48-h light submergence (Light Sub) treatment. Data was normalized according to hydroxylation of LCBs (A) and saturation extends of LCBs (B) in ceramides. Values represent means ±SD (n = 4). *P<0.05 or **P<0.01 by Student’s t-test. (TIF) [file pgen.1005143.s004.tif]

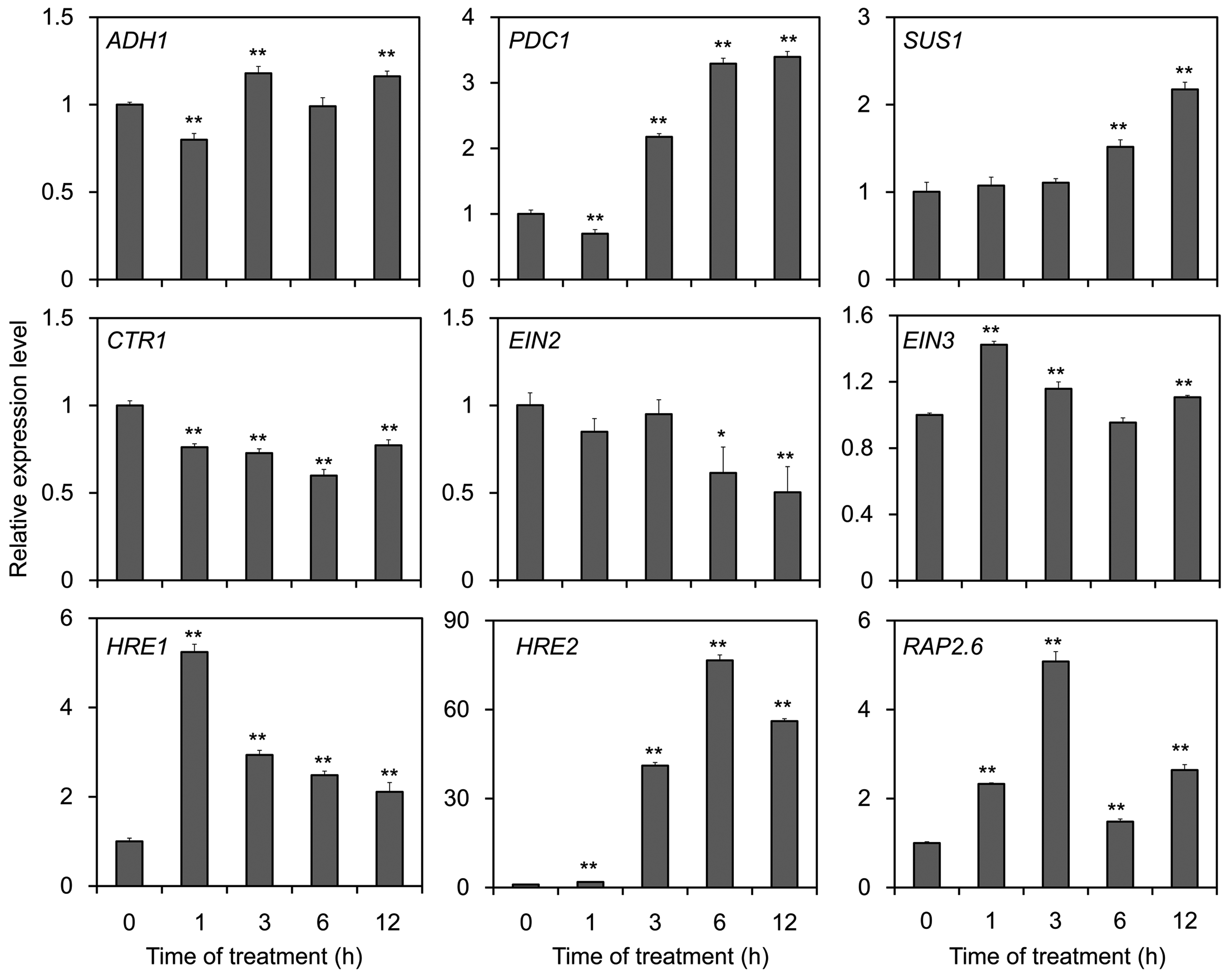

Supplement: S5 Fig — Total RNA was isolated from 2-week-old wild type (WT) seedlings treated with ceramides by floating on MS liquid medium containing 0.1 mM ceramide liposomes (Cer 24:1). The samples were collected at 0, 1, 3, 6 and 12 h after treatment and the relative expression levels of hypoxia responsive genes (ADH1, PDC1, SUS1, CTR1, EIN2, EIN3, HRE1, HRE2 and RAP2.6) were determined by real-time PCR analysis. Expression levels of each time point were normalized to both 0 h and ACTIN2. The experiments have been repeated and similar results were obtained. Values represent means ±SD (n = 3).*P<0.05 or **P<0.01 by Student’s t-test. (TIF) [file pgen.1005143.s005.tif]

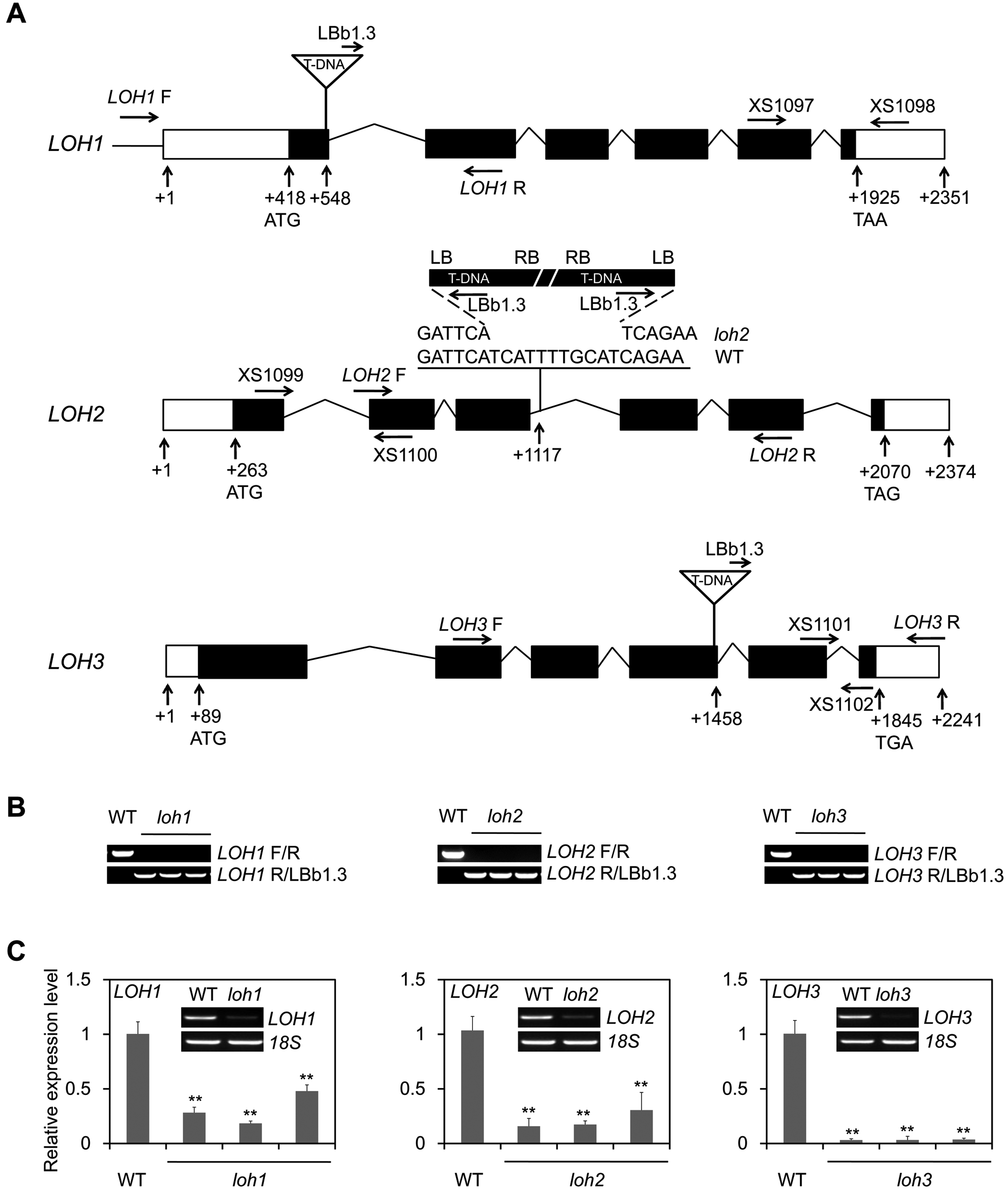

Supplement: S6 Fig — (A) T-DNA insertion sites of the knockout mutants within LOH1 (AT3G25440), LOH2 (AT3G19260) and LOH3 (AT1G13580) genes. Primers designed for genotyping are indicated. White and black boxes indicate UTRs and exons, respectively. Lines between the black boxes indicate introns. (B) Genotyping of the loh1, loh2 and loh3 mutants by PCR. Genomic DNA extracted from wild type (WT), loh1, loh2 and loh3 mutants was amplified using the primer pairs indicated on the right. (C) RT-PCR (electrophoretogram) and qRT-PCR (column chart) analyses showing the knockout or knockdown transcriptions of LOH genes in the loh1, loh2 and loh3 mutants, respectively. Total RNAs isolated from WT, loh1, loh2 and loh3 mutants were employed for RT-PCR or qRT-PCR analyses. (TIF) [file pgen.1005143.s006.tif]

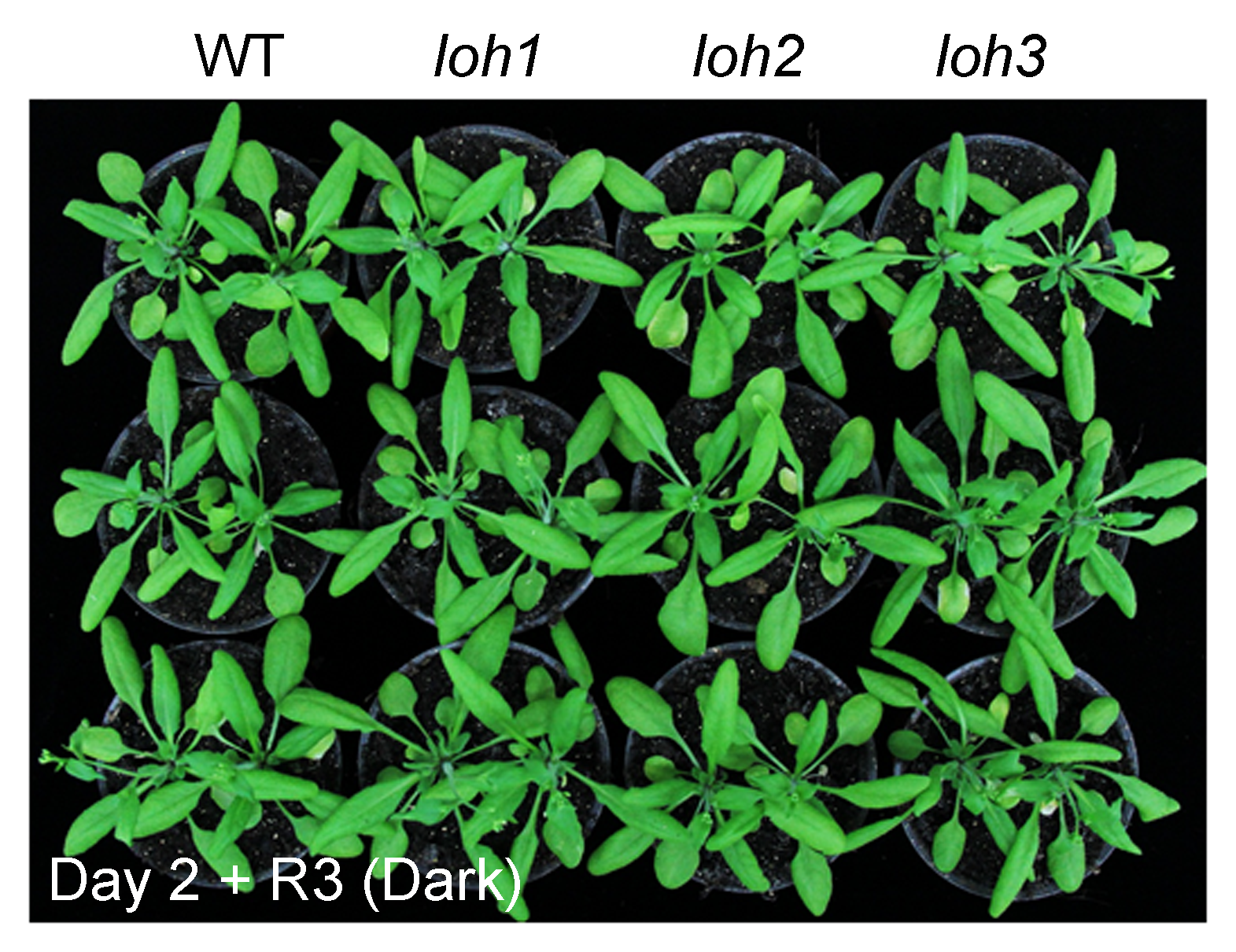

Supplement: S7 Fig — Four-week-old WT, loh1, loh2 and loh3 mutants were placed under constant dark condition for 2 d, and followed by recovery for 3 d. Few phenotypic difference between WT and loh mutants were observed. The experiment has been independently repeated four times with similar results. (TIF) [file pgen.1005143.s007.tif]

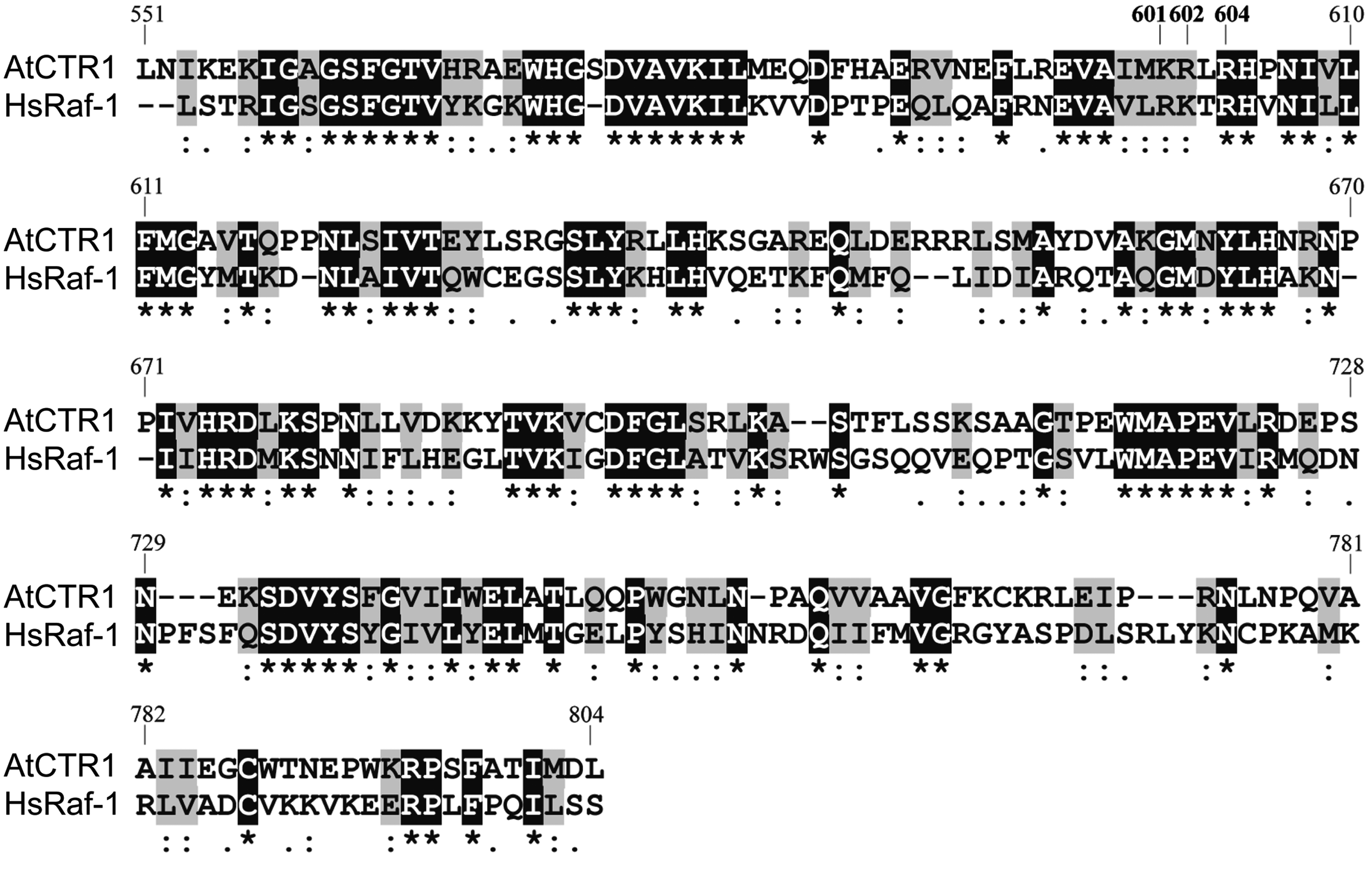

Supplement: S8 Fig — Sequence alignment showing the conserved lipid binding sites K(R)601, R(K)602 and R604 in the kinase domains of Arabidopsis CTR1 and human Raf-1 proteins. (TIF) [file pgen.1005143.s008.tif]

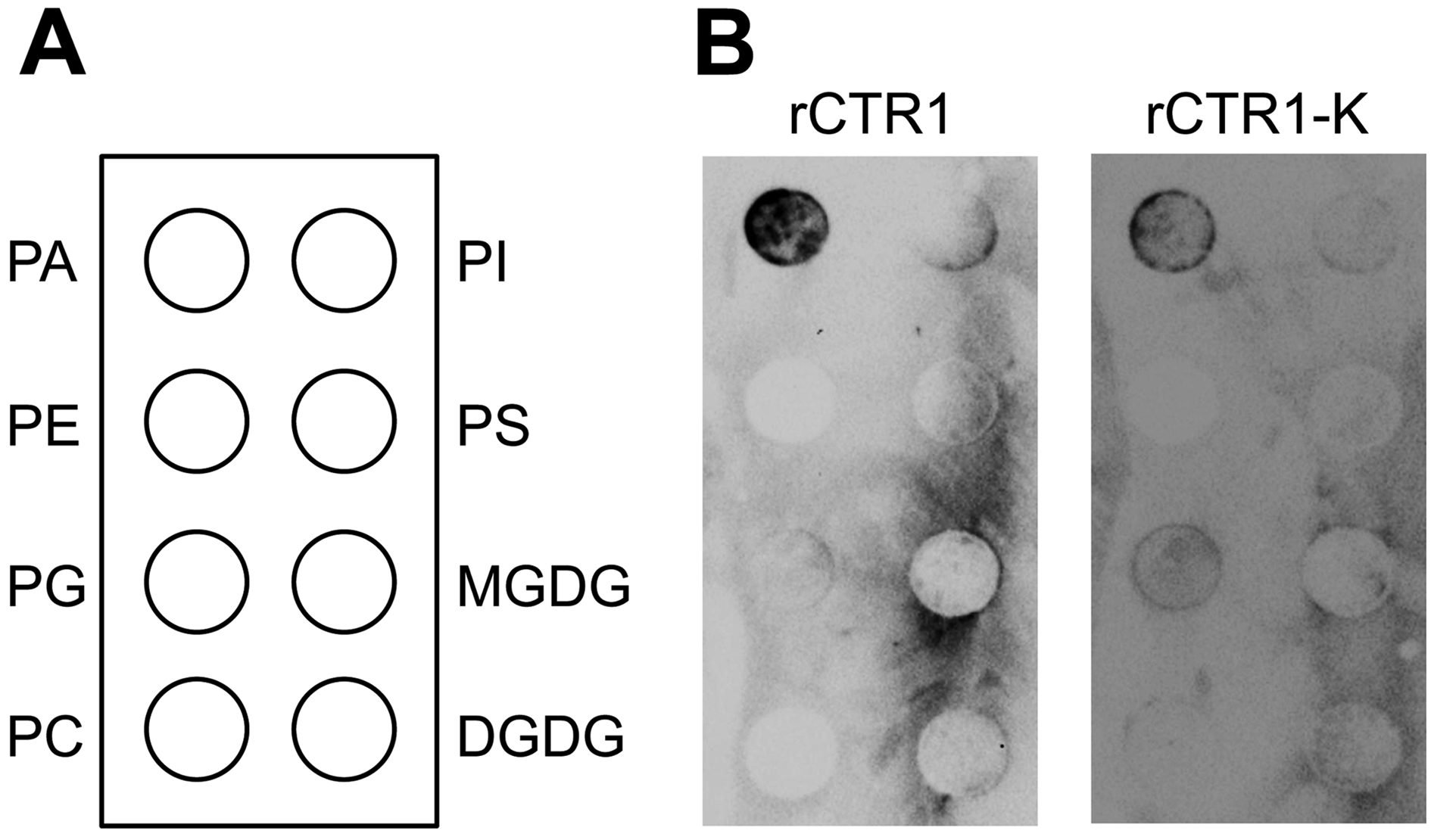

Supplement: S9 Fig — Twenty-five micromole concentrations of various lipids (PA, PG, PE, PC, PI, PS, MGDG and DGDG) were spotted onto nitrocellulose (A) and incubated with 1 μg/mL of either purified rCTR1 or rCTR1-K protein. The interaction between proteins and lipids was detected by immunoblotting using anti-GST antibodies (for rCTR1) or anti-His antibodies (for rCTR1-K) (B). (TIF) [file pgen.1005143.s009.tif]
