# Supplementary material for: Unsaturation of Very-Long-Chain Ceramides Protects Plant from Hypoxia-Induced Damages by Modulating Ethylene Signaling in Arabidopsis
Source: PLoS Genet. 2015 Mar 30;11(3):e1005143. doi: 10.1371/journal.pgen.1005143 (PMC4379176; doi:10.1371/journal.pgen.1005143)
Supplement: S6 Table — (DOC) [file pgen.1005143.s015.doc]

**S6_Table. Sequence of Primers Used in This Study.**

| Name   |  | | --- | |  | | Sequence （5'-3'） | Usage |
| --- | --- | --- | --- | --- |
| ACTIN-F | CCCGCTATGTATGTCGC | qRT-PCR for *ACTIN2* |
| ACTIN-R | AAGGTCAAGACGGAGGAT |
| ADH1-F | TATTCGATGCAAAGCTGCTGTG | qRT-PCR for *ADH1* |
| ADH1-R | CGAACTTCGTGTTTCTGCGGT |
| PDC1-F | CGATTATGGCACTAACCGGATT | qRT-PCR for *PDC1* |
| PDC1-R | TGTTCACCACCGCCTGATAAC |
| SUS1-F | ACGCTGAACGTATGATAACGCG | qRT-PCR for *SUS1* |
| SUS1-R | AACCCTGGAAAGCAAGGCAAG |
| XS1105 | TGAGACCTCAGCTAGGGTTTATC | qRT-PCR for *EIN2* |
| XS1106 | TAAGTGCATGCGCAACTCCCAC |
| XS1107 | GCATGTCCACATCGAGACAGTCG | qRT-PCR for *EIN3* |
| XS1108 | GAGTTCACTGGCCTTGGCTGAG |
| XS1111 | CTACGCTTTCTGCGGCGGCT | qRT-PCR for *CTR1* |
| XS1112 | GTCTGCTGCGCCCAGCTCTT |
| HRE1-F | GATTGATACACAATGGCTCGAA | qRT-PCR for *HRE1* |
| HRE1-R | CGAGAAATATTCGGTCTGGTTC |
| XS1039 | GAAGCGTAAACCCGTCTCAGT | qRT-PCR for *HRE2* |
| XS1040 | TTTGCTCGGGCACGAATCT |
| HUP09-F | TCATCGGCGGACATAGCAA | qRT-PCR for *HUP09* |
| HUP09-R | ATCATCAACCACCCAACCTCC |
| HB1-F | TTTGAGGTGGCCAAGTATGCA | qRT-PCR for *HB1* |
| HB1-R | TGATCATAAGCCTGACCCCAA |
| LBD41-F | TGAAGCGCAAGCTAACGCA | qRT-PCR for *LBD41* |
| LBD41-R | ATCCCAGGACGAAGGTGATTG |
| RAP2.12-F | CGCTGAGTTCGAAGCTGATT | qRT-PCR for *RAP2.12* |
| RAP2.12-R | GTTCCAAGCCAGATTCTAGC |
| RAP2.6-F | GCTGTGACTAAAGAATGTGAAAGC | qRT-PCR for *RAP2.6* |
| RAP2.6-R | CCTTGTGTGGGTCTCGAATCTC |
| XS1097 | TCCGATTCTGAAAGCGATGATG | qRT-PCR for *LOH1* |
| XS1098 | ATTCCTAGTCTCCGTGTGGTT |
| XS1099 | GGATTCTTCTTCTTGAGGCTTGTC | qRT-PCR for *LOH2* |
| XS1100 | CCGAGTAGCAGCATCATTCAAT |
| XS1101 | CTCTCCTATATTGCTTGCTTGTTCT | qRT-PCR for *LOH3* |
| XS1102 | AATCAGTCTTCGTGCTCATCTTC |
| 18S-F | GCTCGAAGACGATCAGATACC | qRT-PCR for *18S* |
| 18S-R | AGAAAGAGCTCTCAGTCTGTC |
| XS1181 | CTCGAGAATATAAAAGAAAAGATTGGAGCAG | For constructing pRSET A-CTR1-K |
| XS1182 | GAATTCCAAATCCGAGCGGTTGGGCGGAGG |
| XS1483 | CCGGAATTCATGGAAATGCCCGGTAGAAGATC | for constructing pGEX-6p-1-CTR1 |
| XS1484 | CCGCTCGAGTTACAAATCCGAGCGGTTGGGCG |
| 1F | TTCACCTGCTTTATTGATGGG | For identifying *loh1* mutant |
| 1R | CCAAATCTGATCTCCAGGACC |
| 2F | CCAGGAGTTCAATGCTTCAAC | For identifying *loh2* mutant |
| 2R | ATGCTGCTTGTGACTTTTTCG |
| 3F | GACTTGGCCTGATCAACAAAC | For identifying *loh3* mutant |
| 3R | GTTCCATGAGTTGGTCCAATG |
| LBb1.3 | ATTTTGCCGATTTCGGAAC | For identifying *loh1*, *loh2* and *loh3* mutants |
| 35S | CAATCCCACTATCCTTCGCAAGACC | For identifying 35S:EIN3-GFP transgenic plant |
| E3-R | TTAGAACCATATGGATACATCTTGCTGCT |
